# The Mediator CDK8-Cyclin C complex modulates Dpp signaling in *Drosophila* by stimulating Mad-dependent transcription

**DOI:** 10.1371/journal.pgen.1008832

**Published:** 2020-05-28

**Authors:** Xiao Li, Mengmeng Liu, Xingjie Ren, Nicolas Loncle, Qun Wang, Rajitha-Udakara-Sampath Hemba-Waduge, Stephen H. Yu, Muriel Boube, Henri-Marc G. Bourbon, Jian-Quan Ni, Jun-Yuan Ji

**Affiliations:** 1 Department of Molecular and Cellular Medicine, College of Medicine, Texas A&M University Health Science Center, Bryan, Texas, United States of America; 2 School of Medicine, Tsinghua University, Beijing, China; 3 Centre de Biologie Intégrative, Centre de Biologie du Développement, UMR5544 du CNRS, Université de Toulouse, Toulouse, France; 4 Department of Nutrition, Texas A&M University, College Station, Texas, United States of America; University of Colorado Medical School, UNITED STATES

## Abstract

Dysregulation of CDK8 (Cyclin-Dependent Kinase 8) and its regulatory partner CycC (Cyclin C), two subunits of the conserved Mediator (MED) complex, have been linked to diverse human diseases such as cancer. Thus, it is essential to understand the regulatory network modulating the CDK8-CycC complex in both normal development and tumorigenesis. To identify upstream regulators or downstream effectors of CDK8, we performed a dominant modifier genetic screen in *Drosophila* based on the defects in vein patterning caused by specific depletion or overexpression of CDK8 or CycC in developing wing imaginal discs. We identified 26 genomic loci whose haploinsufficiency can modify these CDK8- or CycC-specific phenotypes. Further analysis of two overlapping deficiency lines and mutant alleles led us to identify genetic interactions between the CDK8-CycC pair and the components of the Decapentaplegic (Dpp, the *Drosophila* homolog of TGFβ, or Transforming Growth Factor-β) signaling pathway. We observed that CDK8-CycC positively regulates transcription activated by Mad (Mothers against dpp), the primary transcription factor downstream of the Dpp/TGFβ signaling pathway. CDK8 can directly interact with Mad *in vitro* through the linker region between the DNA-binding MH1 (Mad homology 1) domain and the carboxy terminal MH2 (Mad homology 2) transactivation domain. Besides CDK8 and CycC, further analyses of other subunits of the MED complex have revealed six additional subunits that are required for Mad-dependent transcription in the wing discs: Med12, Med13, Med15, Med23, Med24, and Med31. Furthermore, our analyses confirmed the positive roles of CDK9 and Yorkie in regulating Mad-dependent gene expression *in vivo*. These results suggest that CDK8 and CycC, together with a few other subunits of the MED complex, may coordinate with other transcription cofactors in regulating Mad-dependent transcription during wing development in *Drosophila*.

## Author summary

CDK8 and its dedicated partner CycC are conserved subunits of the Mediator complex that bridges transcription factors with RNA Polymerase II in eukaryotes. Here we explore the function and regulation of the CDK8-CycC pair in *Drosophila* by performing a dominant modifier genetic screen based on wing vein patterning defects caused by specific alteration of CDK8-CycC activities. We have observed that multiple components of the Dpp/TGFβ signaling pathway genetically interact with CDK8-CycC. CDK8 and CycC positively regulate gene expression activated by Mad, the key transcription factor downstream of Dpp/TGFβ signaling, and CDK8 can directly interact with the linker region of the Mad protein. We also identify additional, but not all, subunits of the Mediator complex that play positive roles in regulating Mad-dependent gene expression. Given the fundamental role of Dpp/TGFβ signaling in regulating development and its misregulation in a variety of diseases, understanding how Mad/Smad interacts with the Mediator complex may have broad implications in understanding the pathogenesis of these diseases.

## Introduction

Composed of up to 30 conserved subunits, the Mediator complex plays critical roles in modulating RNA polymerase II (Pol II)-dependent gene expression by functioning as a molecular bridge linking transcriptional activators and the general transcription machinery in almost all eukaryotes [1–5]. Biochemical purification of the human Mediator complex has revealed the Cyclin-Dependent Kinase 8 (CDK8) module, composed of CDK8 (or its paralogue CDK19, also known as CDK8L), CycC, Med12 (or Med12L), and Med13 (or Med13L), and the small Mediator complex, composed of 26 subunits that are divided into the head, middle, and tail modules [6–9]. CDK8 is the only Mediator subunit with enzymatic activities. The CDK8 kinase module (CKM) has been proposed to function in two modes. First, it can reversibly bind with the small Mediator complex to form the large Mediator complex, thereby physically blocking the interaction between the small Mediator complex and the general transcription machinery (notably with RNA Pol II itself). Second, CDK8 can function as a kinase that phosphorylates different substrates, particularly transcriptional activators such as E2F1 [10,11], N-ICD (intracellular domain of Notch) [12], p53 [13], Smad proteins [14,15], SREBP (sterol regulatory element-binding protein) [16], and STAT1 (signal transducer and activator of transcription 1) [17]. These characterized functions of CDK8 highlight fundamental roles of the CKM in regulating transcription.

Besides its roles in specific developmental and physiological contexts, the CKM subunits are dysregulated in a variety of human diseases, such as cancers [18–22]. For example, CDK8 has been reported to act as an oncoprotein in melanoma and colorectal cancers [10,23,24]. Moreover, CDK8 and CDK19 are overexpressed in invasive ductal carcinomas, correlating with shorter relapse-free survival in breast cancer [25]. Gain or amplification of CDK8 activity is sufficient in driving tumorigenesis in colorectal and pancreatic cancers in human, as well as in skin cancer in fish [14,23,26–28]. Because of these discoveries, there is a considerable interest in developing drugs targeting the CDK8 kinase for cancer treatment in recent years [29,30]. However, exactly how CDK8 dysregulation contributes to tumorigenesis remains poorly understood. Thus it is essential to reveal the function and regulation of CDK8 activity in different developmental, physiological, and pathological processes.

The major bottleneck for addressing these critical gaps in our knowledge is the lack of *in vivo* readouts for CDK8-specific activities in metazoans. We overcame this challenge by generating tissue-specific phenotypes caused by varying CDK8 activities in *Drosophila*. After validating the specificity of these phenotypes using genetic, molecular, and cell biological approaches, we performed a dominant modifier genetic screen to identify factors that interact with CDK8 *in vivo* based on these unique readouts for CDK8-specific activities. From the screen, we identified *Dad* (*Daughters against dpp*), which encodes an inhibitory Smad in the Dpp (Decapentaplegic)/TGFβ (Transforming Growth Factor-β) signaling pathway, as well as additional components of the Dpp signaling pathway including *dpp*, *tkv* (*thickveins*, encoding the type I receptor for Dpp), *Mad* (*Mothers against dpp*) and *Medea* (encoding the Smad1/5 and Smad4 homologs, respectively) in *Drosophila*. Consistent with the previous biochemical analyses suggesting that CDK8 may phosphorylate *Drosophila* Mad or human Smad1 [14,15,31], thereby regulating their transcriptional activities [14,15,31], our results have validated and further advanced our understanding of this conserved regulatory mechanism *in vivo*. Furthermore, our analyses have revealed additional Mediator subunits and protein kinases involved in regulating the Mad/Smad-dependent transcription. These results, together with previous studies, suggest that concerted recruitment of the Mediator complexes and other cofactors play a pivotal role in regulating Mad/Smad-dependent gene expression, a critical process for TGFβ signaling to function in a variety of biological and pathological contexts.

## Results

### Wing vein patterning defects caused by varying the levels of CDK8, CycC, or both

**Fig 1:**
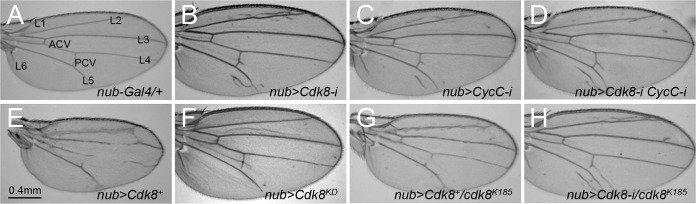
Wing vein patterning defects caused by varying the levels of CDK8, CycC, or both. Adult female wings of (A) *nub-Gal4/+* (control), note the longitudinal veins L1-L6, anterior crossvein (ACV), and posterior crossvein (PCV); (B) *w*^*1118*^*/+; nub-Gal4/+; UAS-Cdk8-RNAi/+*; (C) *w*^*1118*^*/+; nub-Gal4/+; UAS-CycC-RNAi/+*; (D) *w*^*1118*^*/+; nub-Gal4/+; UAS-Cdk8-RNAi CycC-RNAi/+*; (E) *w*^*1118*^*/+; nub-Gal4>UAS-Cdk8*^*+*^*/+*; (F) *w*^*1118*^*/+; nub-Gal4/UAS-Cdk8*^*KD*^; (G) *w*^*1118*^*/+; nub-Gal4>UAS-Cdk8*^*+*^*/+; cdk8*^*K185*^; and (H) *w*^*1118*^*/+; nub-Gal4/+; UAS-Cdk8-RNAi/cdk8*^*K185*^.

To study the function and regulation of CDK8 and CycC *in vivo*, we have generated transgenic lines to either deplete them by RNAi (RNA interference) or conditionally overexpress the wild-type CDK8 kinase using the Gal4-UAS system [32,33]. Normal *Drosophila* wings display stereotypical vein patterns, consisting of six longitudinal veins, dubbed L1 to L6, and two crossveins, the anterior crossvein and the posterior crossvein (Fig 1A). Knocking down of CDK8 using the *nub-Gal4* (*nubbin-Gal4*) line (see Materials and Methods for details), which is specifically expressed in the wing pouch area of the wing imaginal discs [34], results in the formation of ectopic veins in the intervein region, especially around L2 and L5 (Fig 1B). Similar phenotypes were observed with the depletion of CycC (Fig 1C) or both CDK8 and CycC (Fig 1D). In contrast, overexpression of wild-type CDK8 (*UAS-Cdk8*^*+*^) disrupts the L3 vein, the L4 vein, and the crossveins (Fig 1E), opposite to the phenotypes caused by depleting CDK8, CycC, or both. However, overexpression of a kinase-dead (KD) form of CDK8 (*UAS-Cdk8*^*KD*^) using the same approach does not affect the vein patterns (Fig 1F), suggesting that the effects of CDK8 on vein phenotypes are dependent on the kinase activity of CDK8. These observations show that CDK8 and CycC are involved in regulating the vein patterning in *Drosophila*.

**Fig 2:**
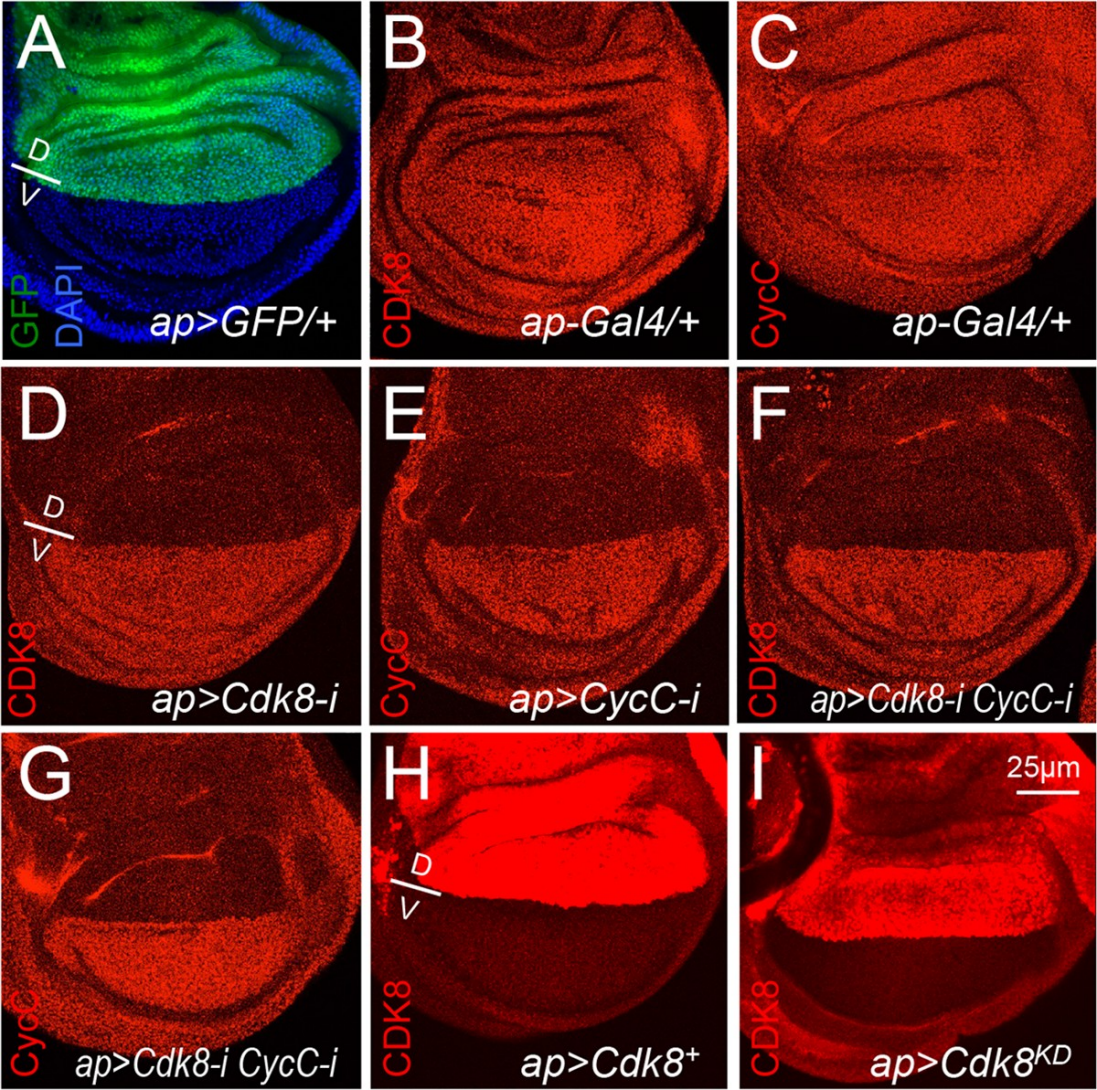
Validation of the specificity of the vein defects caused by depletion or overexpression of CDK8-CycC. Representative confocal images of the wing pouch area of a L3 wandering larval wing disc: (A) *ap-Gal4/UAS-2XGFP* with DAPI (blue) and GFP (green); (B) *ap-Gal4/+* with anti-CDK8 (red) staining; (C) *ap-Gal4/+* with anti-CycC (red) staining; (D) *ap-Gal4/+; UAS-Cdk8-RNAi/+* with anti-CDK8 (red) staining; (E) *ap-Gal4/+; UAS-CycC-RNAi/+* with anti-CycC (red) staining; (F) *ap-Gal4/+; UAS-Cdk8-RNAi CycC-RNAi/+* with anti-CDK8 (red) staining; (G) *ap-Gal4/+; UAS-Cdk8-RNAi CycC-RNAi/+* with anti-CycC (red) staining; (H) *ap-Gal4/UAS-Cdk8*^*+*^ with anti-CDK8 (red) staining; and (I) *ap-Gal4/UAS-Cdk8*^*KD*^ with anti-CDK8 (red) staining. Note that the gain for confocal imaging in H and I is lower than the others to avoid over saturation of the signals. At least five wing discs were examined for each genotype. The dorsal/ventral (D/V) boundary is shown in A, D and H. Scale bar in I: 25μm.

Interestingly, depletion of CDK8 (Fig 1B), CycC (Fig 1C), or both (Fig 1D) increase the size of the wings, correlating to a significant increase of total cell numbers but a reduction of cell sizes (S1 Fig). In contrast, overexpression of wild-type CDK8 reduces the size of wings and total cell numbers, but no obvious effects on cell size (Fig 1E and S1 Fig). The effects of CDK8 on wing size can also be visualized using *ap-Gal4* (*apterous-Gal4*), which is specifically expressed within the dorsal compartment of the wing discs (Fig 2A) [35]. *Ap-Gal4*-induced depletion of CDK8 and CycC caused the adult wing to curl downwards (S2C Fig), indicating the overgrowth of the dorsal compartment compared to the ventral compartment; while overexpression of CDK8 led to the adult wing to curl upwards (S2D Fig), suggesting reduced growth of the dorsal compartment. We have previously reported that CDK8 inhibits the transcriptional activity of E2F1, a key transcription factor that controls the expression of factors required for the G1 to S-phase transition of the cell cycle [10,11]. Thus, the effects of CDK8 levels on wing size and cell numbers are likely through E2F1-regulated cell-cycle progression.

### Validation of the specificity of the vein defects caused by depletion or overexpression of CDK8-CycC

To verify the specificity of these phenotypes, we recombined the *nub-Gal4* line with the CDK8-RNAi, CycC-RNAi, or CDK8-overexpression lines, and then tested whether these vein phenotypes could be dominantly modified by *cdk8*^*K185*^, a null allele of *cdk8* [36]. As shown in Fig 1G, reducing CDK8 by half in a ‘*cdk8*^*K185*^*/+*’ heterozygous background suppresses the vein defects caused by CDK8 overexpression. However, heterozygosity of *cdk8*^*K185*^ does not obviously enhance the vein phenotype caused by CDK8-RNAi (Fig 1H), indicating that the RNAi of CDK8 may have depleted most of the CDK8 protein pool.

To further validate the specificity of the CDK8-directed phenotypes at the cellular level, we analyzed the protein levels of CDK8 and CycC in wing discs at the third instar wandering larval stage by immunostaining with CDK8 or CycC specific antibodies. Normally, both the CDK8 (Fig 2B) and CycC (Fig 2C) proteins are uniformly distributed in the nuclei of all wing disc cells. Depletion of CDK8 (Fig 2D), CycC (Fig 2E), or both (Fig 2F and 2G) using the *ap-Gal4* line significantly reduced CDK8 or CycC proteins in the dorsal compartment. The ventral compartment of the same discs serves as the internal control. In contrast, overexpression of either wild-type (Fig 2H) or kinase-dead (Fig 2I) CDK8 using *ap-Gal4* specifically increased the levels of CDK8 protein in the dorsal compartment. Taken together, these genetic and cell biological analyses have validated the specificity of both the antibodies and transgenic lines, demonstrating that these vein phenotypes are caused by a specific gain or reduction of CDK8 activity *in vivo*.

### Identification of deficiency lines that can dominantly modify the vein phenotypes caused by varying CDK8

Based on these CDK8-specific vein phenotypes, we performed a dominant modifier genetic screen to identify gene products that can functionally interact with CDK8 *in vivo*. The idea of using phenotypic modifications to identify multiple genes involved in determining a specific trait or a phenotypic endpoint was initially developed by Calvin B. Bridges, when he analyzed mutant genes that could interact with the *eosin* mutant in regulating eye color in flies [37]. This genetic modifier approach has been employed to reveal the functional and inter-molecular networks for proteins of interest in *Drosophila* (for instances, [38–42]), and to provide insights into the phenotypic and genetic variability in mammals [43,44]. The approach posits that if a protein interacts with CDK8-CycC *in vivo* in defining the wing vein patterns, then reducing its level by half may either enhance or suppress the sensitized wing vein phenotypes caused by specific alteration of the CDK8 activities. Accordingly, we can survey through the fly genome to search for factors that interact with CDK8-CycC using single genetic crosses.

To facilitate this screen approach, we generated three stocks with the following genotypes: “*w*^*1118*^*; nub-Gal4; UAS-Cdk8-RNAi*” (designated as “*nub>Cdk8-i*” for simplicity), “*w*^*1118*^*; nub-Gal4; UAS-CycC-RNAi*” (“*nub>CycC-i*”), and “*w*^*1118*^*; nub-Gal4*, *UAS-Cdk8*^*+*^*/CyO*” (“*nub>Cdk8*^*+*^”). We then conducted a dominant modifier genetic screen by crossing these three lines in parallel with a collection of 490 deficiency (*Df*) lines (S1 Table), which uncovers the majority of the euchromatic genome [45,46]. Any alteration of the wing vein patterns can be readily discerned under dissecting microscopes, allowing us to search for *Df* lines that could modify the vein phenotypes caused by specific alteration of CDK8 activities.

**Fig 3:**
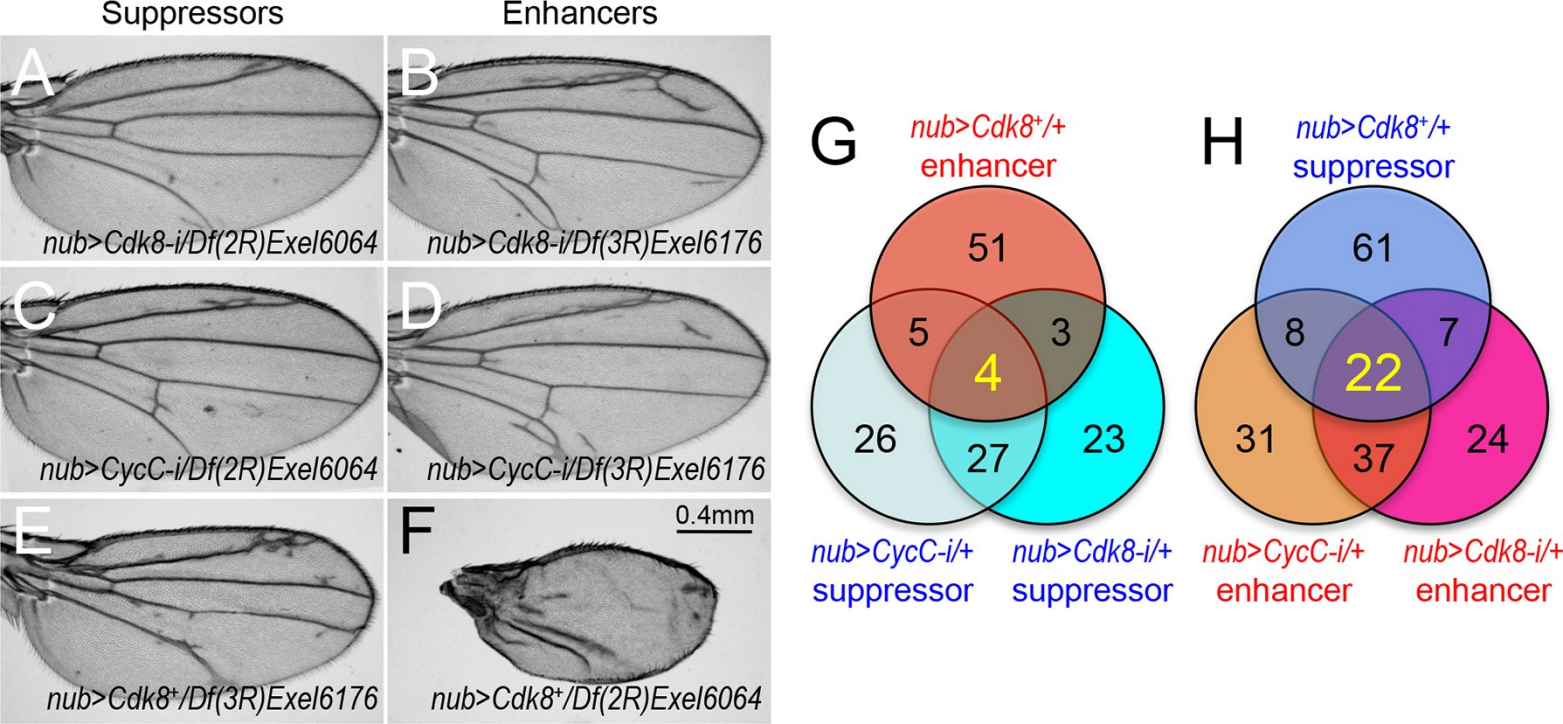
Identification of deficiency lines that can dominantly modify the vein phenotypes caused by varying CDK8. (A-F) Adult wings showing the examples of dominant modifiers. (A) *nub-Gal4/Df(2R)Exel6064; UAS-Cdk8-RNAi* (a suppressor of the CDK8-RNAi phenotype); (B) *nub-Gal4/+; UAS-Cdk8-RNAi/Df(3R)Exel6176*, (an enhancer of the CDK8-RNAi phenotype); (C) *nub-Gal4/Df(2R)Exel6064; UAS-CycC-RNAi/+* (a suppressor of the CycC-RNAi phenotype); (D) *nub-Gal4/+; UAS-CycC-RNAi/Df(3R)Exel6176* (an enhancer of the CycC-RNAi phenotype); (E) *nub-Gal4>UAS-Cdk8*^*+*^*/+; Df(3R)Exel6176 /+* (a suppressor of the CDK8-overexpression phenotype); and (F) *nub-Gal4>UAS-Cdk8*^*+*^*/Df(2R)Exel6064* (an enhancer of the CDK8-overexpression phenotype). Scale bar in F: 0.4mm. (G and H) The Venn diagrams summarize the numbers of suppressors and enhancers of the CDK8-specific phenotypes.

We inspected the vein patterns of the F1 females for enhancers and suppressors based on the following criteria: suppressors of the CDK8- or CycC-RNAi phenotypes are expected to display fewer or no ectopic veins (e.g., Fig 3A and 3C), while enhancers of the CDK8- or CycC-RNAi phenotypes show more or longer ectopic veins (e.g., Fig 3B and 3D). To score the strength of the modifications, we define strong suppressors as the *Df* lines that eliminate all of the ectopic veins, while the *Df* lines that only shorten the length of the ectopic veins are scored as weak suppressors. Similarly, we define strong enhancers to cause more or longer ectopic veins than CDK8- or CycC-RNAi phenotypes, while the *Df* lines causing less severe vein defects are designated as the weak enhancers. Conversely, the strong suppressors of the CDK8-overexpression phenotype are expected to have vein patterns similar to those of wild-type wings (particularly the L3/L4; e.g., Fig 3E, compared to the control shown in Fig 1E). If the *Df* lines only partially restore the missing veins, then they are scored as the weak suppressors. In contrast, the strong enhancers of the CDK8-overexpression phenotype are defined by further disrupting the vein patterns, with the entire L3 or L4 missing, often accompanied with strong disruption on other veins (e.g., Fig 3F); while the weaker enhancers further disrupted the vein defects compared to the CDK8-overexpression phenotype, but less severe than the strong enhancers.

From these screens, we identified 57 suppressor and 90 enhancer *Df* lines for the CDK8-RNAi phenotype, and 62 suppressor and 98 enhancer *Df* lines for the CycC-RNAi phenotype. In addition, we identified 63 enhancer and 98 suppressor *Df* lines for the CDK8-overexpression phenotype (Fig 3G and 3H). The results for all of these *Df* lines are summarized in S1 Table. Of these dominant modifier *Df* lines, four of them suppressed the CDK8-RNAi and CycC-RNAi phenotypes but enhance the CDK8-overexpression phenotype (Fig 3G, Table 1), while 22 of them enhance the CDK8-RNAi and CycC-RNAi phenotypes but suppress the CDK8-overexpression phenotype (Fig 3H, Table 1). To further validate this genetic approach, we generated a transgenic line that allowed us to simultaneously deplete CDK8 and CycC (“*w*^*1118*^*; nub-Gal4; UAS-Cdk8-RNAi*, *CycC-RNAi*”, referred to as “*nub>Cdk8-i CycC-i*”) with *nub-Gal4*, and observed identical phenotypes to the ones caused by depleting either *Cdk8* or *CycC* alone (Fig 1D). With the exception of one *Df* line, the rest of these 25 *Df* lines have consistently modified the ectopic vein phenotype caused by depletion of both CDK8 and CycC: four of the *Df* lines behaved as suppressors and 21 of them as enhancers (Table 1). These results show that the CDK8-specific vein phenotypes are modifiable and can be used to identify factors that functionally interact with CDK8-CycC *in vivo*.

**Table 1:** Deficiency lines that dominantly modify the CDK8- or CycC-specific phenotypes.

Stock #	Deficiency lines	Cytogenetic breakpoints	*nub>CDK8+* background	*nub>CDK8-RNAi* background	*nub>CycC-RNAi* background	*nub>CDK8-RNAi*, *CycC-RNAi* background
901	*Df(1)svr*	1A1;1B9—10	weak suppressor	strong enhancer	enhancer	lethal
25059	*Df(1)BSC531*	3C3;3E2	enhancer	strong suppressor	strong suppressor	enhancer
3196	*Df(1)Sxl-bt*	6E2;7A6	suppressor	strong enhancer	strong enhancer	strong enhancer
1581	*Df(2L)JS31*	23A3—4;23D	strong suppressor	enhancer	enhancer	enhancer
9718	*Df(2L)BSC244*	32F2;33B6	enhancer	strong suppressor	strong suppressor	strong suppressor
7512	*Df(2L)Exel6030*	33A2—33B3	suppressor	enhancer	enhancer	enhancer
7546	*Df(2R)Exel6064*	53C11;53D11	strong enhancer	suppressor	strong suppressor	suppressor
25430	*Df(2R)BSC597*	58A2;58F1	suppressor	strong enhancer	enhancer	weak enhancer
27352	*Df(2R)BSC780*	60C2;60D14	strong suppressor	strong enhancer	strong enhancer	strong enhancer
7561	*Df(2R)Exel6082*	60C4—60C7	suppressor	enhancer	enhancer	enhancer
25436	*Df(2R)BSC603*	60C7—60D1	strong suppressor	enhancer	enhancer	enhancer
24413	*Df(3L)BSC389*	66C12;66D8	suppressor	enhancer	enhancer	enhancer
27577	*Df(3L)BSC816*	66D9;66D12	weak suppressor	enhancer	strong enhancer	enhancer
26525	*Df(3L)BSC673*	67C7;67D10	suppressor	enhancer	weak enhancer	enhancer
7945	*Df(3L)Exel9011*	76B8;76B9	suppressor	weak enhancer	enhancer	enhancer
2596	*Df(3L)6B-29+Df(3R)6B-29*	81Fa;81Fa	suppressor	enhancer	enhancer	no effects
7623	*Df(3R)Exel6144*	83A6-83B6	strong suppressor	enhancer	enhancer	strong enhancer
9215	*Df(3R)ED5495*	85F16;86C7	enhancer	suppressor	suppressor	weak suppressor
7965	*Df(3R)Exel7310*	86E18;87A1	suppressor	strong enhancer	enhancer	enhancer
7976	*Df(3R)Exel8159*	88A4;88B1	suppressor	enhancer	enhancer	weak enhancer
7655	*Df(3R)Exel6176*	89E11;89F1	strong suppressor	weak enhancer	weak enhancer	weak enhancer
26846	*Df(3R)BSC748*	89E5;89E11	strong suppressor	enhancer	enhancer	enhancer
7657	*Df(3R)Exel6178*	90F4;91A5	suppressor	enhancer	enhancer	enhancer
2352	*Df(3R)X3F*	99D1—2;99E1	suppressor	enhancer	enhancer	lethal
2155	*Df(3R)A113*	100A;3Rt	suppressor	strong enhancer	enhancer	enhancer
7918	*Df(3R)Exel8194*	100A4;100A7	suppressor	weak enhancer	enhancer	strong enhancer

### Identification of *Dad* as an enhancer of the *nub>Cdk8-i* and *nub>CycC-i* phenotypes but a suppressor of the *Cdk8*-overexpression phenotype

**Fig 4:**
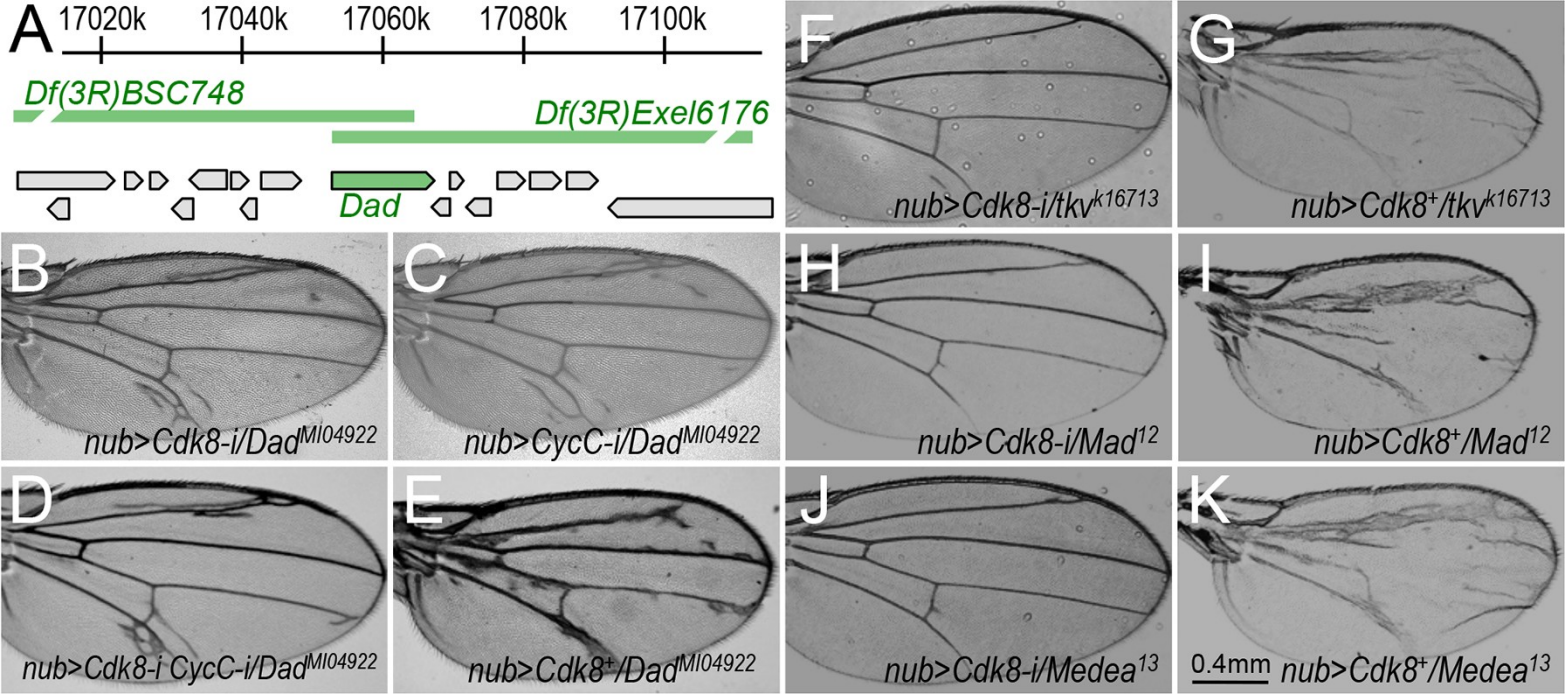
Identification of the *Dad* gene and genes encoding other components of the Dpp signaling pathway as dominant modifiers of the CDK8-specific phenotypes. (A) Schematic diagram of the genome region of *Df(3R)BSC748* and *Df(3R)Exel6176*, which uncover the gene *dad*. Adult wings with the following genotypes: (B) *nub-Gal4/+; UAS-Cdk8-RNAi/Dad*^*MI04922*^; (C) *nub-Gal4/+; UAS-CycC-RNAi/Dad*^*MI04922*^; (D) *nub-Gal4/+; UAS-Cdk8-RNAi CycC-RNAi/Dad*^*MI04922*^; (E) *nub-Gal4>UAS-Cdk8*^*+*^*/+; Dad*^*MI04922*^*/+*; (F) *nub-Gal4/tkv*^*k16713*^*; UAS-Cdk8-RNAi/+*; (G) *nub-Gal4>UAS-Cdk8*^*+*^*/tkv*^*k16713*^; (H) *nub-Gal4/Mad*^*12*^*; UAS-Cdk8-RNAi/+*; (I) *nub-Gal4>UAS-Cdk8*^*+*^*/Mad*^*12*^; (J) *nub-Gal4/+; UAS-Cdk8-RNAi/Medea*^*13*^; and (K) *nub-Gal4>UAS-Cdk8*^*+*^*/+; Medea*^*13*^*/+*; Scale bar in K: 0.4mm.

To identify the specific genes uncovered by these dominant modifier *Df* lines, we analyzed these 26 genome regions with partial overlapping *Df* lines (Table 1). Interestingly, two partially overlapping *Df* lines, *Df(3R)BSC748* and *Df(3R)Exel6176*, enhanced the CDK8-RNAi and CycC-RNAi phenotypes, but suppressed the CDK8-overexpression phenotype (Fig 3B, 3D and 3E; Table 1). The overlapping region uncovers one specific gene, *Dad* (*Daughter against Dpp*), encoding the *Drosophila* homolog of Smad6/7 (Fig 4A). Thus, to test whether *Dad* is the specific gene that accounts for the modification of the CDK8-specific phenotypes by these two *Df* lines, we performed similar genetic tests with two mutant alleles of *Dad*: *Dad*^*MI04922*^, a MiMIC (Minos Mediated Integration Cassette) insertion in an intron of the *Dad* gene [47], and *Dad*^*j1E4*^, an insertion of the *P{lacW}* element in an intron of the *Dad* gene [48]. Indeed, both *Dad* mutant alleles dominantly enhanced the CDK8-RNAi (Fig 4B), CycC-RNAi (Fig 4C), and CDK8-RNAi plus CycC-RNAi (Fig 4D) phenotypes, but suppressed the CDK8-overexpression phenotype (Fig 4E, Table 2). These effects on the CDK8-specific vein phenotypes are similar to those observed for *Df(3R)BSC748* and *Df(3R)Exel6176*, suggesting that *Dad* is the specific gene that genetically interacts with CDK8 *in vivo*.

**Table 2:** Mutant alleles of genes encoding components of the Dpp signaling that modify the CDK8- or CycC-specific phenotypes.

Mutant alleles	*nub>Cdk8*^*+*^	*nub>Cdk8-i*	*nub>CycC-i*	*nub>Cdk8-i CycC-i*
*dpp*^*d6*^	NE	Suppressor	Suppressor	Suppressor
*dpp*^*hr92*^	NE	Suppressor	Suppressor	Suppressor
*dpp*^*S11*^	NE	Suppressor	Suppressor	Suppressor
*tkv*^*7*^	Enhancer	NE	Suppressor	Suppressor
*tkv*^*k16713*^	Enhancer	Suppressor	NE	Suppressor
*Mad*^*k00237*^	Enhancer	NE	Suppressor	Suppressor
*Mad*^*1-2*^	NE	Suppressor	NE	Suppressor
*Mad*^*8-2*^	NE	Suppressor	NE	Suppressor
*Mad*^*12*^	Enhancer	Suppressor	NE	NE
*Mad*^*KG00581*^	Enhancer	NE	NE	Suppressor
*Medea*^*1*^	Enhancer	NE	Suppressor	Suppressor
*Medea*^*13*^	Enhancer	Suppressor	No Effect	Suppressor
*Dad*^*MI04922*^	Suppressor	Enhancer	Enhancer	Enhancer
*Dad*^*j1e4*^	Suppressor	Enhancer	Enhancer	Enhancer
NE, no effects.					

### Mutants of multiple components of the Dpp signaling pathway genetically interact with CDK8-CycC

The protein Dad functions as an inhibitory Smad in the Dpp/TGFβ signaling pathway, which plays critical roles in regulating cell proliferation and differentiation during the development of metazoans [49–54]. During the development of the wing discs, Dpp spreads from the anterior-posterior boundary to the anterior and posterior halves [49–51,55]. Upon the binding of the Dpp ligand to the Tkv-Punt receptor complex on the cell membrane, the TGFβ type II receptor Punt phosphorylates and activates the type I receptor Tkv. This results in the phosphorylation of Mad by Tkv at its C-terminal SSXS motif, known as the phospho-Mad protein or pMad. Medea, the unique co-Smad protein in *Drosophila*, associates with pMad in the cytoplasm, and then this heteromeric Smad complex translocates into the nucleus and regulates the expression of its target genes [53,55–57].

The genetic interactions between CDK8-CycC and Dad prompted us to test whether mutant alleles of other components of the Dpp signaling pathway could also genetically interact with CDK8 and CycC. For this, we crossed multiple mutant alleles of these components with the CDK8-CycC depletion or overexpression lines. As summarized in Table 2, mutants of multiple components of the Dpp signaling pathway could either dominantly enhance or suppress the CDK8-specific vein phenotypes. For instance, *dpp*^*d6*^, *dpp*^*hr92*^, *dpp*^*S11*^, *tkv*^*7*^, *tkv*^*k16713*^ (Fig 4F), *Mad*^*1-2*^, *Mad*^*12*^ (Fig 4H), *Mad*^*8-2*^, *Mad*^*k00237*^, *Mad*^*KG00581*^, *Medea*^*1*^, and *Medea*^*13*^ (Fig 4J) all dominantly suppress the ectopic vein phenotype caused by depletion of CDK8, CycC, or both CDK8 and CycC (Table 2). However, *tkv*^*7*^, *tkv*^*k16713*^ (Fig 4G), *Mad*^*k00237*^, *Mad*^*12*^ (Fig 4I), *Mad*^*KG00581*^, *Medea*^*1*^, and *Medea*^*13*^ (Fig 4K) enhance the CDK8-overexpression phenotype (Table 2). Testing additional mutant alleles of these genes have revealed that most of them can also dominantly modify the CDK8-specific phenotypes (Table 2). Dpp is activated in a specific pattern in the middle part of the wing pouch area, while the *nub-Gal4* display a well-characterized pattern in the entire wing pouch area. These two patterns differ, arguing against the possibility that Dpp signaling may affect *nub-Gal4* expression pattern. In addition, reducing Mad or Dad by half has little effects on the expression of a *UAS-RFP* reporter driven by *nub-Gal4* (S3 Fig), suggesting that the expression and activity of *nub-Gal4* are not affected by Dpp signaling. Taken together, these genetic interactions suggest that CDK8-CycC may affect vein patterning by modulating Dpp signaling.

### CDK8-CycC positively regulates Mad-dependent transcription

Given that CDK8 and CycC are known subunits of the Mediator complex, which serves as a scaffold complex mediating the interactions between the RNA Pol II basal transcription machinery and a number of gene-specific transcription activators [3,7,58]. Thus, the simplest model to explain the genetic interactions between Dpp signaling and CDK8-CycC is that the CDK8-CycC complex may directly regulate the transcriptional activity of Mad in the nucleus. To test this model, we analyzed the effects of CDK8-CycC depletion on the expression of *salm* (*spalt major*), a well-characterized direct target gene of Mad involved in vein differentiation [59–62]. The *sal-lacZ* (*P{PZ}salm*^*03602*^) is a enhancer trap line derived from an insertion of a *P{PZ}* element in the promoter region of the *salm* gene [63,64], and the expression of *sal-lacZ* can serve as a reporter for the transcriptional activity of Mad [65].

**Fig 5:**
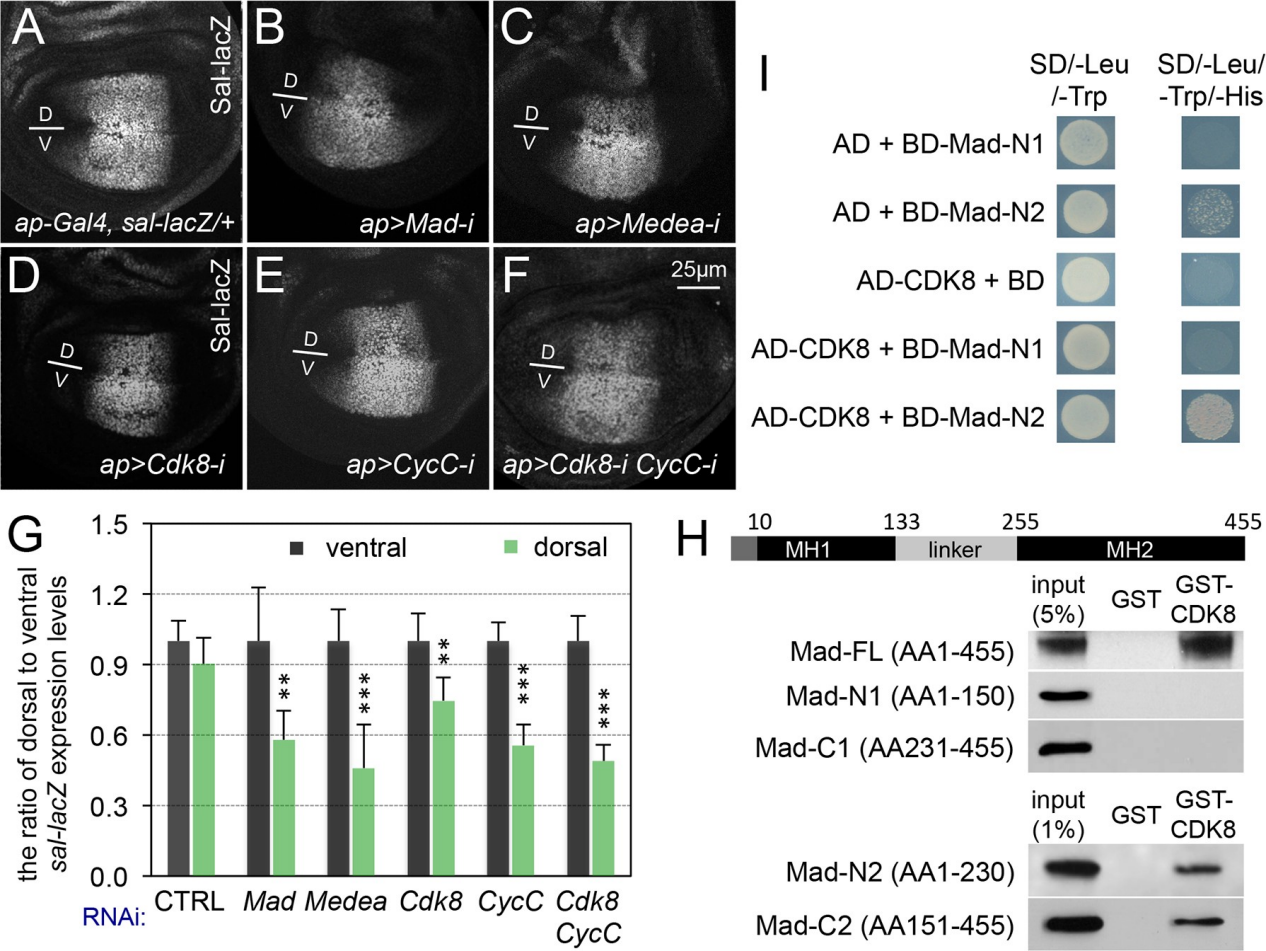
CDK8-CycC positively regulates Mad-dependent transcription. Confocal images of the wing pouch area of a L3 wandering larval wing disc of (A) *ap-Gal4*, *sal-lacZ/+* (control); (B) *ap-Gal4*, *sal-lacZ/UAS-Mad-RNAi* (BL-43183); (C) *ap-Gal4*, *sal-lacZ/UAS-Medea-RNAi*; (D) *ap-Gal4*, *sal-lacZ/+; UAS-Cdk8-RNAi/+*; (E) *ap-Gal4*, *sal-lacZ/+; UAS-CycC-RNAi/+*; and (F) *ap-Gal4*, *sal-lacZ/+; UAS-Cdk8-RNAi CycC-RNAi/+*. All signals presented were from anti-β-galactosidase staining. Scale bar in F: 25μm. Dorsal (D)-ventral (V) boundaries are marked using a short line in these images. (G) Quantification of Sal-lacZ expression. The black columns represent the average of Sal-lacZ expression in the ventral compartment of the indicated genotypes (N = 5 for each genotype), and light green columns represent the measurements in the dorsal compartments. (H) Western Blots of a GST pull-down assay between GST-CDK8 and His-tagged Mad fragments. The amino acids (AA) positions of MH1 and MH2, separated by the linker region, are based on a BLAST search of *Drosophila* Mad-RA isoform (455AA). The other isoform, Mad-RB (525AA), has additional 70AA at the N-terminus. We focused on the Mad-RA isoform in this work. (I) Y2H assay showing the specific interaction between CDK8 and the linker region of Mad. SD/-Leu/-Trp and SD/-Leu/-Trp/-His are synthetic dropout (SD) media lacking “Leu and Trp”, or “Leu, Trp, and His”, respectively. The co-transformed yeast cultures were spotted on SD/-Leu/-Trp and SD/-Leu/-Trp/-His plates, positive interactions result in yeast growth on the SD/-Leu/-Trp/-His plate. AD, GAL4-activation domain (prey); BD, GAL4-DNA-binding domain (bait); AD- or BD-protein, AD- or BD-fusion proteins.

Because the expression of *sal-lacZ* is symmetric along the dorsal-ventral boundary of the wing pouch area of the wing discs (Fig 5A), we tested whether specific depletion of CDK8 or CycC within the dorsal compartment of the wing discs could affect the transcriptional activity of Mad by detecting the transcription level of *sal* using an anti-β-galactosidase (anti-β-Gal) antibody. For this, we depleted genes of interest using the *ap-Gal4* driver, and then compared the β-Gal expression between the dorsal and ventral compartments. As expected, depleting Mad with two transgenic RNAi lines (BL-43183 (Fig 5B) and BL-31315 (S4B and S4B’ Fig)), Medea (Fig 5C), or Dpp (S4A Fig) using this approach reduced the expression of the *sal-lacZ* reporter in the dorsal compartment. Importantly, depletion of CDK8 (Fig 5D), CycC (Fig 5E), or both (Fig 5F), in the dorsal compartment significantly decreased the β-Gal expression level in the dorsal compartment compared with the ventral compartment of the same disc. After quantifying the line-scan profiles of the Sal-lacZ levels in the wing porch area, we calculated the relative signal intensity of dorsal to ventral Sal-lacZ levels for 5 wing discs of each genotype (S5 Fig; see Materials and Methods for details), which validated the effects of CDK8-CycC on *sal-lacZ* expression (Fig 5G). Similar observations were made by quantification of the pixel intensities in areas in the dorsal and ventral compartments (S6 Fig).

**Fig 6:**
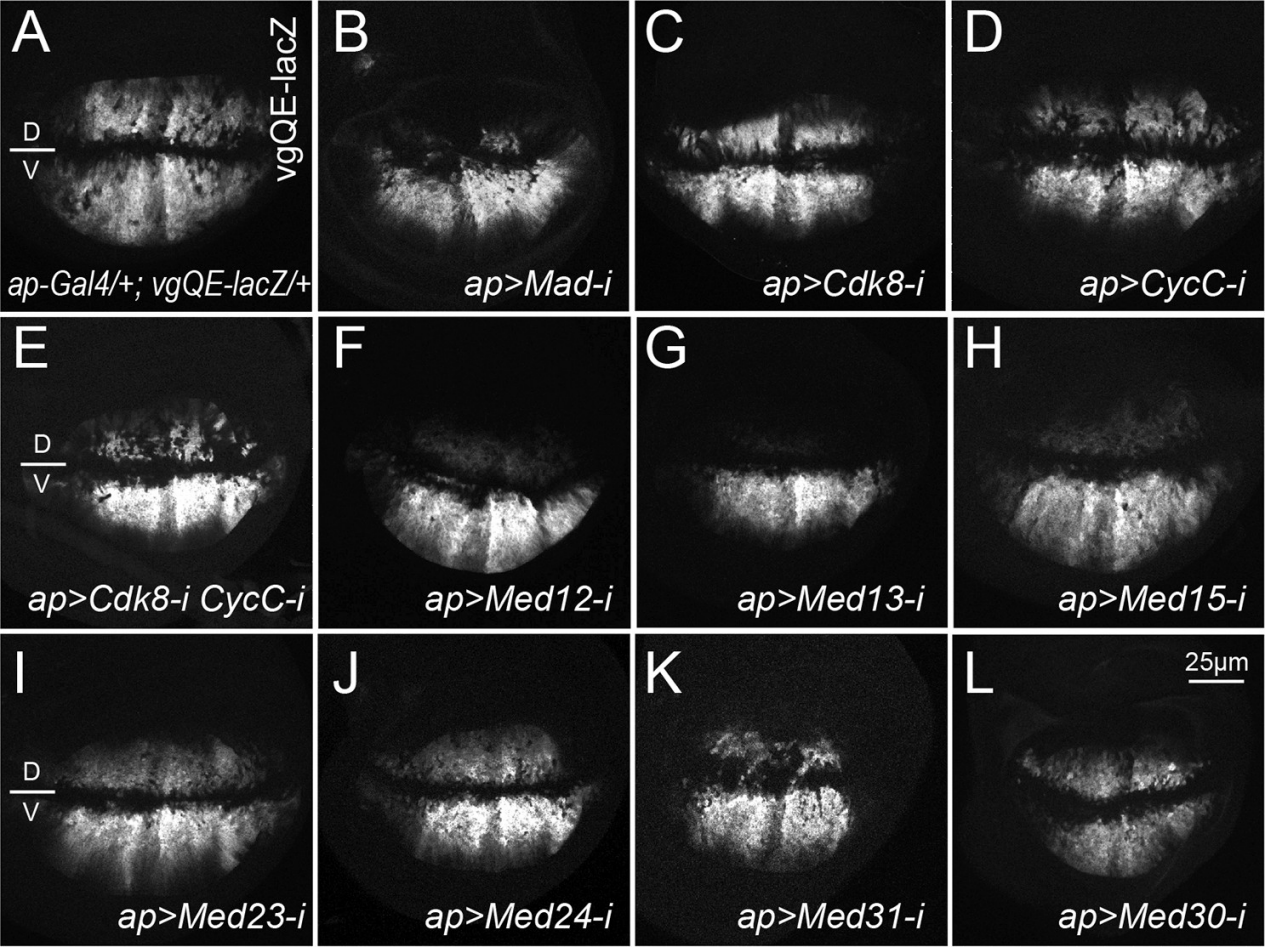
Effects of various Mediator subunits on the expression of the *vgQE-lacZ* reporter. Representative confocal images of anti-β-Gal staining of wing discs of the following genotypes: (A) *ap-Gal4/+; vgQE-lacZ/+;* (B) *ap-Gal4/+; vgQE-lacZ/UAS-Mad-RNAi* (BL-31315); (C) *ap-Gal4/+; vgQE-lacZ/UAS-Cdk8-RNAi*; (D) *ap-Gal4/+; vgQE-lacZ/UAS-CycC-RNAi*; (E) *ap-Gal4/+; vgQE-lacZ/UAS-Cdk8-RNAi CycC-RNAi*; (F) *ap-Gal4/+; vgQE-lacZ/UAS-Med12-RNAi*; (G) *ap-Gal4/+; vgQE-lacZ/UAS-Med13-RNAi*; (H) *ap-Gal4/+; vgQE-lacZ/UAS-Med15-RNAi*; (I) *ap-Gal4/+; vgQE-lacZ/UAS-Med23-RNAi*; (J) *ap-Gal4/+; vgQE-lacZ/UAS-Med24-RNAi*; (K) *ap-Gal4/+; vgQE-lacZ/UAS-Med31-RNAi*; (L) *ap-Gal4/+; vgQE-lacZ/UAS-Med30-RNAi*. At least five wing discs were examined for each genotype. Scale bar in L: 25μm.

To further validate the effects of CDK8-CycC depletion on Mad-activated gene expression, we analyzed the expression of the quadrant enhancer (QE) of the selector gene *vestigial* (*vgQE-lacZ*) in wing discs. Similar to *Sal-lacZ* reporter, *vgQE-lacZ* also displays a symmetric expression pattern along the D-V boundary in the wing pouch (Fig 6A) [66,67]. As expected, depleting Mad (BL-31315) using *ap-Gal4* driver significantly reduced the expression of *vgQE-lacZ* in the dorsal compartment (Fig 6B). Although depleting CDK8 alone only marginally reduced the *vgQE-lacZ* expression in the dorsal compartment (Fig 6C), a more obvious effect was observed with the depletion of CycC (Fig 6D), and a stronger reduction of the reporter expression was detected with the depletion of both CDK8 and CycC (Fig 6E) using the same approach. We note that the interpretation of the data presented in Fig 6 is compounded by the fact that the transcription of the *vg* in different compartments of wing discs is controlled by Wingless (Wg) and Dpp signaling, as well as a feed-forward regulation by Vg itself [66,67]. Nevertheless, the most parsimonious model to explain the observations based on *Sal-lacZ* and *vgQE-lacZ* reporters is that CDK8-CycC positively regulates Mad-dependent transcription.

One caveat of these analyses is that the CKM could affect *ap-Gal4* activities. As shown in S7B Fig, we observed that depleting CDK8 and CycC reduces the *ap-Gal4*-dependent expression of *UAS-GFP* in the dorsal compartment of wing discs (compared to the control shown in S7A Fig). This observation suggests that the positive effects of depletion of CDK8 and CycC on wing vein patterning are hypomorphic, representing an under-estimation of the positive effects of CDK8-CycC in regulating Mad-dependent transcription. In addition, we observed that depleting Ap protein using *ap-Gal4* has no effects on the *sal-lacZ* expression in the dorsal compartment (S7C Fig), suggesting that the expression of *sal-lacZ* is independent of the levels of Ap or Gal4.

### Direct interactions between CDK8 and Mad

Since Mad phosphorylation at its C-terminus (pMad) by the Tkv-Punt receptor complex marks the activation of Mad, we tested whether CDK8 affects the pMad level. For this, we depleted CDK8, CycC, or both, with the *ap-Gal4* line, and then detected the levels of the activated Mad with an anti-pMad antibody. In the wing pouch area of the control discs, the pMad protein is symmetrically distributed along the dorsal-ventral boundary (S8A Fig). However, depletion of CDK8-CycC did not affect pMad levels when comparing the dorsal compartment with the ventral compartment (S8B–S8D Fig), suggesting that CDK8-CycC does not affect the phosphorylation of Mad at its carboxy terminus in the cytoplasm. These results support the idea that the CDK8-CycC complex directly regulates the transcriptional activity of Mad in the nucleus.

R-Smads are characterized by a highly conserved amino-terminal MH1 (Mad homology 1) domain that binds to DNA and a C-terminal MH2 (Mad homology 2) domain that harbors the transactivation activity, separated by a serine- and proline-rich linker region (Fig 5H) [68]. It was previously reported that CDK8 and a few other kinases (see below) may phosphorylate Smad proteins in both *Drosophila* and mammalian cells [14,15,31,55,68], but whether and how CDK8 interacts with Smads remain unknown. To determine whether CDK8 directly interacts with Mad, we performed a GST-pulldown assay. As shown in Fig 5H, purified GST-CDK8 can directly bind with His-tagged full length Mad (Mad-FL, AA1-455) expressed in *E*. *coli*. We then mapped the specific domain of Mad that interacts with CDK8 using His-tagged fragments of the Mad protein. We observed that the “Mad-N2” fragment (AA1-230) and the “Mad-C2” fragment (AA151-455), but not the “Mad-N1” fragment (AA1-150) or the “Mad-C1” fragment (AA231-455), can interact directly with CDK8 (Fig 5H). We validated the interaction between CDK8 and the linker region using the yeast two-hybrid (Y2H) assay: the “Mad-N2” fragment, but not the “Mad-N1” fragment, as the bait can interact with full-length CDK8 as the prey (Fig 5I). It is not feasible to use this Y2H approach test with Mad-FL or Mad-C1/C2 fragments as bait, since they auto-activate as the baits; while the full-length CDK8 can also auto-activate as the bait (S9 Fig). Taken together, these results suggest that CDK8 directly interacts with part of the linker region of Mad protein (AA151-230). Implications of these physical interactions are discussed below.

### Involvement of additional Mediator complex subunits in regulating the Mad/Smad-dependent transcription

**Fig 7:**
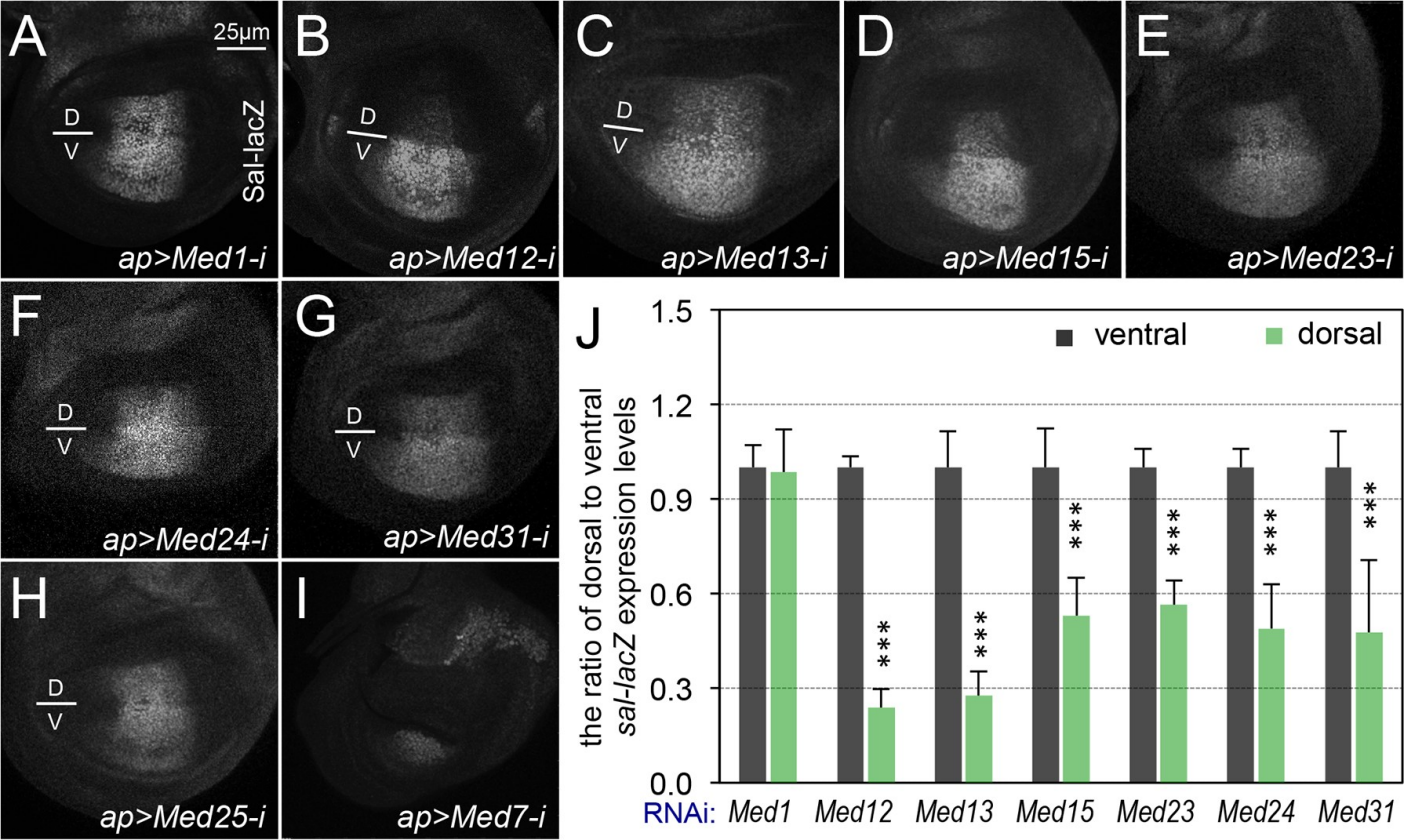
Effects of the additional Mediator subunits on the expression of the *sal-lacZ* reporter. Representative confocal images of anti-β-Gal staining of wing discs of the following genotypes: (A) *ap-Gal4*, *sal-lacZ/+; UAS-Med1-RNAi/+*; (B) *ap-Gal4*, *sal-lacZ/+; UAS-Med12-RNAi/+*; (C) *ap-Gal4*, *sal-lacZ/+; UAS-Med13-RNAi/+*; (D) *ap-Gal4*, *sal-lacZ/+; UAS-Med15-RNAi/+*; (E) *ap-Gal4*, *sal-lacZ/+; UAS-Med23-RNAi/+*; (F) *ap-Gal4*, *sal-lacZ/+; UAS-Med24-RNAi/+*; (G) in *ap-Gal4*, *sal-lacZ/+; UAS-Med31-RNAi/+*; (H) *ap-Gal4*, *sal-lacZ/UAS-Med25-RNAi*; and (I) *ap-Gal4*, *sal-lacZ/+; UAS-Med7-RNAi/+*. (J) Quantification of Sal-lacZ expression. The black columns represent the average of Sal-lacZ expression in the ventral compartment of five wing discs of the indicated genotypes (N = 5 for each genotype), and light green columns represent the measurements in the dorsal compartments. Scale bar in A: 25μm. For (H) and (I), at least five wing discs were examined for each genotype.

The Med15/ARC105 subunit of the Mediator complex has been previously shown to directly interact with the transactivation MH2 domain of Smad2/3, thereby mediating the Smad2/3-Smad4-dependent transcription in *Xenopus* [69], and Med15 is required for the transcription of Dpp target genes in *Drosophila* [70]. However, whether other Mediator subunits are involved in regulating the Mad/Smad-dependent transcription remains unknown. To address this question, we depleted individual subunits of the Mediator complex upon conditional expression of interfering RNAs with *ap-Gal4*, and then analyzed the expression of the *sal-lacZ* reporter. Of the 30 Mediator subunits tested (Table 3), we have observed that depletion of six additional Mediator subunits, Med12 (Fig 7B), Med13 (Fig 7C), Med15 (Fig 7D), Med23 (Fig 7E), Med24 (Fig 7F), and Med31 (Fig 7G), by *ap-Gal4* significantly reduced the expression of *sal-lacZ* in cells of the dorsal compartment compared with the cells in the ventral compartment of the same wing discs (Fig 7J); similar to depletion of CDK8 or CycC (Fig 5). The effects of these six Mediator subunits were further validated using the *vgQE-lacZ* reporter: their depletion using *ap-Gal4* also reduces the *vgQE-lacZ* expression in the dorsal compartment (Fig 6F–6K). These results suggest that these Mediator subunits are required for the Mad-activated gene expression. However, RNAi depletion of the remaining 15 Mediator subunits using *ap-Gal4* driver did not significantly affect *sal-lacZ* expression (Table 3), as β-Gal expression remained symmetric along the dorsal-ventral boundary as exemplified for depletion of Med1 (Fig 7A and 7J) and Med25 (Fig 7H) on *sal-lacZ* expression. Similarly, depletion of Med30 using *ap-Gal4* does not obviously affect the expression of *sal-lacZ* and *vgQE-lacZ* reporters, which remains symmetric along the dorsal-ventral boundary (Fig 6L, Table 3). Furthermore, depleting the remaining Mediator subunits, including Med7 (Fig 7I), Med8 (S10A Fig), Med14 (S10B Fig), Med16 (S10C Fig), Med17 (S10D Fig), Med21 (S10E Fig), and Med22 (S10F Fig), severely disrupted the morphology of the wing discs, making it difficult to determine their roles in regulating *sal* transcription. Taken together, these observations suggest that multiple Mediator subunits, but apparently not all of them, are required for Mad-dependent transcription in *Drosophila*.

**Table 3:** The effects of depleting different Mediator subunits on the expression of the *sal-lacZ* and *vgQE-lacZ* reporters in wing discs during the third instar larval stage, as well as the wing and eye phenotypes in adult flies.

Mediator subunit	Effect on *sal-lacZ* expression	Effect on *vgQE-lacZ* expression	Phenotypes using the *nub-Gal4* driver	Phenotypes using the *ey-Gal4* driver	Terriente-Felix et al. (2010) (*nub-Gal*4)
CDK8	decrease	decrease	ectopic vein	NE	ND
CycC	decrease	decrease	ectopic vein	NE	ND
CDK8 & CycC	decrease	decrease	ectopic vein	NE	ND
Med12	decrease	decrease	pupal lethal	small eye	small wing
Med13	decrease	decrease	pupal lethal	small eye	ND
Med15	decrease	decrease	cell death	small eye	ND
Med23	decrease	decrease	vein defects	NE	ND
Med24	decrease	decrease	NE	small eye	ND
Med31	decrease	decrease	cell death	pupal lethal	ND
Med7	deformed	ND	wingless	pupal lethal	ND
Med8	deformed	ND	pupal lethal	pupal lethal	ND
Med14	deformed	ND	wingless	pupal lethal	ND
Med16	deformed	ND	pupal lethal	small eye	small wing
Med17	deformed	ND	wingless	pupal lethal	ND
Med21	deformed	deformed	pupal lethal	pupal lethal	ND
Med22	deformed	ND	cell death	eyeless	ND
Med1	NE	ND	cell death	NE	ND
Med4	NE	ND	vein defects	NE	ND
Med6	NE	ND	larval lethal	small eye	ND
Med9	NE	ND	NE	NE	ND
Med10	NE	ND	NE	NE	small wing
Med11	NE	ND	cell death	eyeless	ND
Med18	NE	ND	NE	NE	ND
Med19	NE	ND	ectopic vein	NE	ND
Med20	NE	ND	vein defects	small eye	cell death
Med25	NE	ND	small wing	NE	small wing
Med26	NE	ND	NE	small eye	ND
Med27	NE	ND	cell death	eyeless	small wing
Med28	NE	ND	cell death	small eye	ND
Med29	NE	ND	NE	NE	ND
Med30	NE	NE	cell death	small eye	cell death
NE, no effects.					
ND, not determined.					

### CDK9 and Yorkie also positively regulate the Mad/Smad-dependent transcription

**Fig 8:**
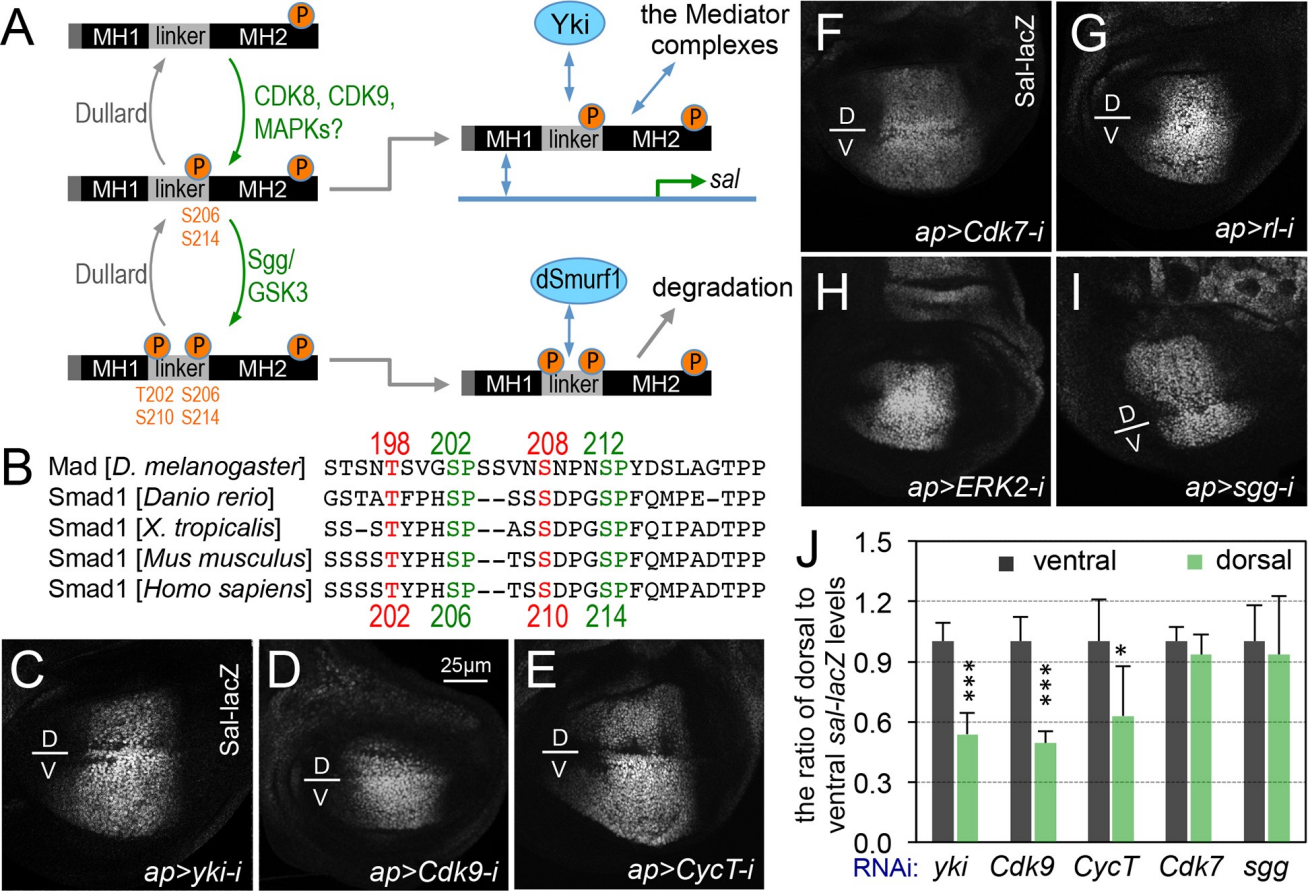
Validation of additional transcriptional cofactors for their roles in regulating Mad-dependent transcription. (A) Model: linker region of pMad may be phosphorylated by CDK8, CDK9, or MAPKs as priming kinase recruiting Yki/YAP binding to pMad to drive target gene, such as *sal* transcription; and further phosphorylation by Sgg/GSK3 at the linker region may switch the binding to dSmuf1 and causes pMad degradation. (B) Sequence alignment of part of the Mad/Smad1 linker region from different species showing the conservation of the potential phosphorylation sites by CDKs, MAPKs, and GSK3. Representative confocal images of anti-β-Gal staining of wing discs of the following genotypes: (C) *ap-Gal4*, *sal-lacZ/+; UAS-yki-RNAi/+*; (D) *ap-Gal4*, *sal-lacZ/UAS-Cdk9-RNAi*; (E) *ap-Gal4*, *sal-lacZ/+; UAS-CycT-RNAi/+*; (F) *ap-Gal4*, *sal-lacZ/UAS-Cdk7-RNAi*; (G) *ap-Gal4*, *sal-lacZ/+; UAS-rl-RNAi/+*; (H) *ap-Gal4*, *sal-lacZ/+; UAS-ERK2-RNAi/+*; and (I) *ap-Gal4*, *sal-lacZ/UAS-sgg-RNAi*. Scale bar in D: 25μm. (J) Quantification of Sal-lacZ expression. The grey columns represent the average of Sal-lacZ expression in the ventral compartment of the indicated genotypes, and light green columns represent the measurements in the corresponding dorsal compartments. N = 5 for the quantification of *sal-lacZ* expression after depleting Yki, Cdk9, or CycT in the dorsal compartment; N = 3 for the quantification of *sal-lacZ* expression after depleting Cdk7 or Sgg in the dorsal compartment. At least five wing discs were examined for depletion of Rl (G) and ERK2 (H), and the represented images were shown.

Besides CDK8, several other kinases, such as CDK7, CDK9, GSK3 (Glycogen synthase kinase 3), and MAPKs (mitogen-activated protein kinases) such as ERK (extracellular signal-regulated kinase) and ERK2, have been implicated to phosphorylate and regulate the transcriptional activity of Smads [14,15,68,71] (Fig 8A, see below). The four phosphorylation sites (Ser or Thr residues) within the linker region of Smads appear to be conserved from *Drosophila* to mammals (Fig 8B; see Discussion). The phosphorylation of Smads within the linker region may facilitate the subsequent binding with transcription co-factors, such as YAP (Yes-associated protein) [14]. However, it is still unclear whether all of these kinases regulate Smads activity *in vivo*. With the exception of YAP (Yorkie or Yki, in *Drosophila*), it is also unclear whether these regulatory mechanisms are conserved during evolution.

To validate the relevance of these kinases in regulating Mad-dependent gene expression, we depleted the *Drosophila* orthologs of CDK7, CDK9, Shaggy (Sgg, the GSK3 homolog in *Drosophila*), Rolled, and dERK2 (MAPK/ERK homologs in *Drosophila*), in the dorsal compartment of wing discs (using *ap*-Gal4 as above), and then analyzed *sal-lacZ* expression in the wing pouch. As expected for a positive role of Yki in regulated Mad-dependent transcription [14], depletion of Yki in the dorsal cells significantly reduced the expression of *sal-lacZ* compared to the cells in the ventral compartment of the same discs (Fig 8C and 8J). Using the same approach, we have observed that depleting CDK9 (Fig 8D and 8J) and its partner CycT (Cyclin T, Fig 8E and 8J; [72]) also reduced *sal-lacZ* expression. These observations suggest that both Yki and CDK9-CycT are required for Mad/Smad-dependent transcription in *Drosophila*, which is consistent to the previous reports [14,31]. However, depletion of CDK7 (Fig 8F and Fig 8J) or *Drosophila* MAPK homologs, either Rolled (Fig 8G) or dERK2 (Fig 8H), did not affect the expression of *sal-lacZ*. Although depletion of Sgg increased the size of the dorsal compartment, the intensity of anti-β-Gal staining remained similar to the ventral compartment (Fig 8I and 8J). We note that depleting CDK9 (S7D Fig), Med12 (S7E Fig), or Med13 (S7F Fig) have no obvious effects on the expression of UAS-GFP reporter, suggesting that their effects on *sal-lacZ* expression are independent of the Gal4 activity *per se*. Together with the previous reports [14,68], our *in vivo* analyses have validated the conserved roles of CDK8-CycC, CDK9-CycT, and Yki/YAP on Mad/Smad-dependent transcription.

## Discussion

To study the function and regulation of CDK8 *in vivo*, we have developed a genetic system that yields robust readouts for the CDK8-specific activities in developing *Drosophila* wings. These genetic tools provide a unique opportunity to perform a dominant modifier genetic screen, allowing us to identify multiple components of the Dpp/TGFβ signaling pathway that can genetically interact with the CDK8-CycC complex *in vivo*. Our subsequent genetic and cellular analyses reveal that CDK8, CycC, and six additional subunits of the Mediator complex, as well as CDK9 and Yki are required for the Mad-dependent transcription in the wing discs. In addition, CDK8 can directly interact with the linker region of Mad. These results have extended the previous biochemical and molecular analyses on how different kinases and transcription cofactors modulate the Mad/Smad-activated gene expression in the nucleus. Further mapping of specific genes uncovered by other deficiency lines may also open up the new directions to advance our understanding of the conserved function and regulation of CDK8 during development.

### Multiple subunits of the Mediator complex are required for Mad/Smad-dependent transcription

The Mediator complex functions as a molecular bridge between gene-specific transcription factors and the RNA Pol II general transcription apparatus, and diverse transactivators have been shown to interact directly with distinct Mediator subunits [4,6–9,73]. However, it is unclear whether all Mediator subunits are required by different transactivators to regulate gene expression, or whether Mediator complexes composed of fewer and different combinations of Mediator subunits exist in differentiated tissues or developmental stages. Gene-specific combinations of the Mediator subunits may be required in different transcription processes, as not all Mediator subunits are simultaneously required for all transactivation process [74]. For instance, ELK1 target gene transcription requires Med23, but lacking Med23 does not functionally affect some other ETS transcription factors, such as Ets1 and Ets2 [75]. Similarly, Med15 is required for the expression of Dpp target genes, but does not appear to affect the expression of EGFR (epidermal growth factor receptor) and Wg targets in *Drosophila* [70].

It has been previously reported that the Med15 subunit is required for the Smad2/3-Smad4 dependent transcription, as its removal from the Mediator complex abolishes the expression of Smad-target genes and disrupts Smad2/3-regulated dorsal-ventral axis formation in *Xenopus* embryos [69]. Further biochemical analyses showed that increased Med15 enhances, while its depletion decreases, the transcription of Smad2/3 target genes, and that the Med15 subunit can directly bind to the MH2 domain of Smad2 or Smad3 [69]. In *Drosophila*, loss or reduction of Med15 reduced the expression of Dpp targets, resulting in smaller wings and disrupted vein patterning (mainly L2) [70]. We also observed that depletion of Med15 or CDK8 reduces the expression of a Mad-target gene. These observations support the idea that CDK8 and Med15 play a conserved and positive role in regulating Mad/Smad-activated gene expression.

Aside from Med15 and CDK8, it remains unclear whether other Mediator subunits are also involved in Mad/Smad-dependent transcription. We identified six additional Mediator subunits that are required for the Mad-dependent transcription, including CycC, Med12, Med13, Med23, Med24, and Med31 (Fig 5, Fig 6, Fig 7 and Table 3). Interestingly, aside from Med23 and Med24 being specific to metazoans, counterparts of the other six subunits are not essential for cell viability in the budding yeast [5]. The similar effects of the four CKM subunits on Mad-activity suggest that they may function together to stimulate Mad-dependent transcription. We note that depletion of seven Mediator subunits, Med7, Med8, Med14, Med16, Med17, Med21, and Med22, severely disrupts the morphology of the wing discs (Fig 7I and S10 Fig), making it difficult to assay their effects on the transcriptional activity of Mad *in vivo*. Consistently, all corresponding subunits, except Med16, are critical for cell viability in the budding yeast [5]. In contrast, reducing expression of the 15 remaining subunits of the *Drosophila* Mediator complex did not significantly alter the expression of a Mad-dependent reporter (Table 3). Med1 and Med25 are loosely associated to the small Mediator complex in human cell lines [5]. A caveat for these negative results is that depleting these subunits using the existing RNAi lines may not be sufficient to affect *sal-lacZ* expression, even though the majority of these transgenic RNAi lines can generate severe phenotypes in the eye, wing, or both (Table 3). Further analyses are necessary to validate these negative data in the future. Taken together, our results indicate that not all Mediator subunits are required for the expression of the Mad-target genes that we tested in the developing wing discs.

### Role of Yki/YAP and different kinases in regulating Mad/Smad-dependent transcription

Interestingly, Yki/YAP, which can function as a transcriptional co-factor for Mad/Smad, was also reported to associate with several subunits of the Mediator complex to drive transcription. Specifically, Med12, Med14, Med23, and Med24 were identified from a YAP IP-mass spectrometry sample in HuCCT1 cells [76]. Med23 was also reported to regulate Yki-dependent transcription of *Diap1* in wing discs [77]. In this work, we found that Yki, Med12, Med23, and Med24 were also required for Mad-dependent transcription of *sal-lacZ*. Although the exact molecular mechanisms of how Yki interacts with certain Mediator subunits remain unclear, it is plausible that Yki may further strengthen the binding between Mad and Med15 through interactions with other subunits such as Med12, Med23, and Med24.

**Fig 9:**
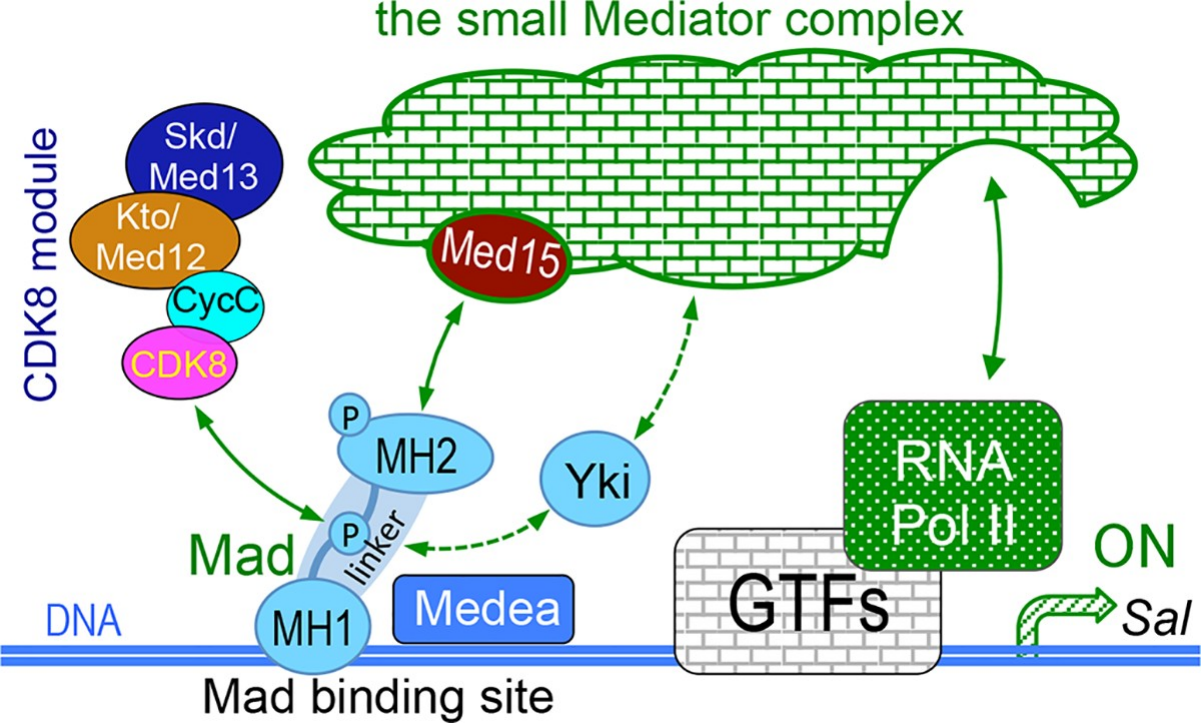
Working model. Model of Mad/Smad-dependent transcription activation through the CKM and the Mediator complex. GTFs, General Transcription Factors; MH1, Mad homology 1; MH2, Mad homology 2.

Based on biochemical analyses of the Smad1 phosphomutants and cell biological analyses using cultured human epidermal keratinocytes (HaCaT cells), several kinases including CDK8, CDK9, and ERK2 were shown to phosphorylate serine residues (Ser, or S) within the linker region of pSmad1 at S186, S195, S206, and S214, or the equivalent sites in pSmad2/3/5. These modifications were proposed to regulate positively Smad1-dependent transcriptional activity [14]. Of these sites, S206 and S214 are both conserved from *Drosophila* to humans (Fig 8B). In addition, studies using *Xenopus* embryos and cultured L cells suggest that MAPKs may phosphorylate the linker region of Smad1 (including S214) and lead to its degradation [71]. Nevertheless, analyses with *Drosophila* embryos and wing discs indicate that S212 (equivalent to human pSmad1 S214) is phosphorylated by CDK8, while S204 (unique in *Drosophila*) and S208 (equivalent to human pSmad1 S210) are phosphorylated by Sgg/GSK3 [15]. These studies suggest the following model in explaining how Smads activate the expression of their target genes and how this process is turned off (Fig 8A, Fig 9): after Smads are phosphorylated at their C-termini and translocated into the nucleus, CDK8 and CDK9 (potentially also MAPKs) act as the priming kinases to further phosphorylate pSmads in the linker region at S206 and S214. This may facilitate the interaction between pSmads and transcriptional cofactors such as YAP, stimulating the expression of Smads target genes. Overexpression of Yki in *Drosophila* wing disc increases the expression of the *vgQE-lacZ* reporter [14], which validates the role of Yki/YAP in activating Mad/Smad1-dependent gene expression *in vivo*. Subsequently, pSmads are further phosphorylated by GSK3 within the linker region at T202 and S210, which may facilitate Smad1/5 binding to E3 ubiquitin ligases such as Smurf1 and Nedd4L, causing the degradation of Smads through the ubiquitin-proteasome pathway [14,15,31,55,68].

Although this model (Fig 9) is still rather speculative, it serves as a conceptual framework to explain how transactivation of Smads is coupled to its degradation, similar to other transcriptional activators [78]. It is challenging to determine whether these kinases act redundantly or sequentially for different phosphorylation sites, the exact orders of these phosphorylation events, as well as their biological consequences *in vivo*. Moreover, it remains unexplored whether these regulatory mechanisms are conserved during evolution. The importance of these issues is highlighted by the critical role of TGFβ signaling in regulating the normal development of metazoans and the dysregulation of this pathway in a variety of human diseases such as cancers [54,79–81].

The precise spatiotemporal activation of the Dpp signaling pathway in the wings discs is critical for proper formation of the stereotypical vein patterns in *Drosophila* [59,62]. This model system provides an ideal opportunity to dissect the dynamic regulation of the Mad-activated gene expression in the nucleus. Indeed, depleting CDK8 in wing discs reduces expression of the Mad-dependent *sal-lacZ* reporter, suggesting that CDK8 positively regulates Mad-dependent transcription. This is consistent with the effects of CDK8 on Smad1/5-dependent transcription in mammals [14,82]. Depleting CDK8 does not affect the phosphorylation of Mad at its C-terminus as revealed by pMad immunostaining (S8 Fig), nor does it affect the physical interaction between CDK8 and the linker region of Mad, supporting the idea that CDK8 may only affect subsequent phosphorylation of Mad, presumably within the linker region.

Besides CDK8-CycC, depleting CDK9-CycT also decreases the expression of the *sal-lacZ* reporter, supporting the notion that CDK8-CycC and CDK9-CycT may play non-redundant roles in further phosphorylating pMad in the nucleus. However, we did not observe any effects of depletion of CDK7 or MAPKs on *sal*-*lacZ* expression, suggesting that their role in regulating the transcriptional activity of Smads may not be conserved in *Drosophila*. Alternatively, the two MAPK/ERK homologs, Rolled and ERK2, may act redundantly in regulating Mad-dependent transcription. Lastly, depleting Sgg/GSK3 in the dorsal compartment of the wing disc increases the size of this compartment, yet the expression level of the *sal-lacZ* reporter is similar to the ventral compartment. These observations are consistent with previous reports that phosphorylations of Mad/Smad in the linker regions by CDK8-CycC and Sgg/GSK3 regulate the level and range of Mad-dependent gene expression [14,15,31,55,68].

Together with the previous reports [14,15,31,55,68,83], our data support that CDK8-CycC and CDK9-CycT may phosphorylate pMad at the linker region, which may facilitate the binding between Yki and Mad. We speculate that this interaction may synergize the recruitment of the Mediator complex, presumably at least through the interaction between its Med15 subunit and the MH2 domain of Mad (Fig 9). Alternatively, Yki may also facilitate the recruitment of the whole Mediator complex through its interactions with Med12, Med23, and Med24. The synergistic interactions among Mad, Yki, the Mediator complex, and RNA Pol II may be required for the optimal transcriptional activation of the Mad-target genes (Fig 9).

One of the challenges is to illustrate the dynamic interactions between these factors and diverse protein complexes that couple the transactivation effects of Mad/Smads on gene transcription with their subsequent degradation at the molecular level. Smad3 phosphorylation strongly correlates with Med15 levels in breast and lung cancer tissues; together, they potentiate metastasis of breast cancer cells [84]. Thus, it will be important to test whether additional Mediator subunits that we identified in *Drosophila* play similar roles in mammalian cells. It will also be interesting to determine whether a partial Mediator complex, composed of a subset of the Mediator subunits, exists and regulates Mad/Smad-dependent gene expression. Furthermore, detailed biochemical analyses may yield mechanistic insights into how CDK8 and Med15 act in concert in stimulating the Mad/Smad-dependent gene expression.

### Potential role of CDK8-CycC in regulating cross-talks among different signaling pathways

Wing pouch-specific alteration of CDK8 activity results in two major phenotypes: disrupted vein patterns and altered size of wing blades. While the effects on wing size and cell numbers can be explained by the role of CDK8 in regulating cell proliferation through E2F1 [10,11], the effects of CDK8 on vein patterning are more complex. The stereotypical wing vein patterns in adult flies are gradually defined by elaborated spatiotemporal interplays among different signaling pathways, including Dpp, EGFR, Hedgehog (Hh), Notch (N), and Wingless (Wg), in the developing wing discs [55,59,60,62]. During the larval and pupal stages, these signaling pathways and their downstream transcriptional targets coordinately control the cell proliferation and differentiation of cell in different parts of the wing disc to form individual veins.

It is noteworthy that varying CDK8 activities has different effects on different veins: gain of CDK8 causes the loss of the L3 and L4 veins, but the vein patterns of L2 and L5 appear thicker and more diffusive; while the ectopic veins caused by reduction of CDK8 are mainly intertwined with the L2 and L5 veins (Fig 1). Our analyses on the genetic interactions between CDK8 and the components of the Dpp signaling pathway led us to discover the role of the Mediator complex in Mad-stimulated transcription of *sal*. However, there is a gap in our understanding of how reduced expression of *sal* in wing discs is linked to the vein defects in adult wings. It is known that *salm* and *salr* (*spalt-related*), two members of the *spalt* gene family that encode zinc-finger transcriptional repressors, function downstream of the Dpp signaling pathway during development of the central part of the wing [85]. Depletion of either *salm* or *salr* alone resulted in ectopic vein formation around L2 in adult wings, yet depletion or loss of both *salm* or *salr* caused loss of vein phenotype [61,86]. In addition, elimination of L2 in ventral-anterior and ectopic L5 in dorsal-posterior were observed in *salm/salr* clones at different region of the wing [61]. These observations suggest that the dosage of *salm* and *salr* in wing discs does not have a linear relationship with the wing vein patterning at the adult stage.

Interestingly, it is known that the CKM complex regulates the transcriptional activities of the key transcription factors of these pathways, including N-ICD downstream of N signaling [12], Mad/Smad proteins ([14,15] and this work). In addition, Med12 (Kohtalo, or Kto in *Drosophila*) and Med13 (Skuld, or Skd in *Drosophila*) subunits of the CKM interact with Pangolin (the lymphoid-enhancing factor (LEF)/T cell factor (TCF) homolog in *Drosophila*), the key transcription factor downstream of Wg signaling, through the transcriptional cofactors such as Pygopus, Legless, and Armadillo [87]. In mammalian cells, Med12 is also known to regulate the activities of Gli proteins, the key transcription factors downstream of Hh signaling [88,89]. Furthermore, the Mediator subunit Med23 interacts with ETS (E-twenty six transcription factor) proteins, a family of key transcription factors downstream of the EGFR signaling pathway [75]. However, whether CDK8-CycC also regulates TCF-, ETS- or Gli-dependent transcription is still not understood. Nevertheless, these studies in other biological contexts suggest that the effects of CDK8 on wing vein patterning are not likely solely through the Dpp signaling pathway. Therefore, we speculate that the potential interactions between CDK8 and the aforementioned signaling pathways may contribute to these differential effects on distinct veins. Further analyses of these cross-talks, as well as further mapping of other *Df* lines that modify the CDK8-specific vein phenotypes, may yield the insights into the molecular and dynamic mechanisms underlying these vein phenotypes.

### Identification of novel genomic loci that genetically interact with CDK8 *in vivo*

To understand how dysregulated CDK8-CycC contributes to a variety of human cancers, it is essential to elucidate the function and regulation of CDK8 *in vivo*. Given that the CDK8-CycC pair and other subunits of the Mediator complex are conserved in almost all eukaryotes [5], *Drosophila* serves as an ideal model system to identify both the upstream regulators and the downstream effectors of CDK8 activity *in vivo*. Our dominant modifier genetic screen is based on the wing vein phenotypes caused by specific alteration of CDK8 activity in the developing wing disc, which serves as a unique *in vivo* readout for the CDK8-specific activities in metazoans. This screen led us to identify 26 genomic regions that include loci whose haplo-insufficiency could consistently modify CDK8-CycC depletion or CDK8-overexpression phenotypes. Identification of *Dad* and genes encoding additional components of the Dpp signaling pathway provides a proof of principle for this approach. Since each of the chromosomal deficiencies uncovers multiple genes, further mapping of the relevant genome regions is expected to identify the specific genetic loci encoding factors that may function either upstream or downstream of CDK8 *in vivo*. It is hoped that further analyses of the underlying molecular mechanisms in both *Drosophila* and mammalian systems will advance our understanding of how dysregulation of CDK8 contributes to human diseases, thereby aiding the development of therapeutic approaches.

## Materials and methods

### Fly strains

Flies were raised on a standard cornmeal, molasses and yeast medium, and all genetic crosses were maintained at 25˚C. The *UAS-Cdk8*^*+*^ and *UAS-Cdk8*^*KD*^ lines were generated using the pUASt vector [36]. The construct allowing conditional expression of a kinase-dead CDK8 form (D173A; [90]) was generated through site-specific mutagenesis by double PCR, using the overlap extension method. The *UAS-Cdk8-RNAi* and *UAS-CycC-RNAi* lines were generated using the pVALIUM20 vector [91], and the *UAS-Cdk8-RNAi CycC-RNAi* line was generated using the pNP vector [92]. The *vgQE-lacZ* line was received from Gary Struhl [66,67].

We obtained the following strains from the Bloomington *Drosophila* Stock Center: *ap-Gal4* (BL-3041), *nub-Gal4* (BL-25754), *sal-lacZ* (BL-11340), *UAS-Cdk7-RNAi* (BL-57245), *UAS-Cdk9-RNAi* (BL-34982), *UAS-CycT-RNAi* (BL-32976), *UAS-dpp-RNAi* (BL-33618), *UAS-2xEGFP* (BL-6874), *UAS-erk-RNAi* (BL-34744), *UAS-Mad-RNAi* (BL-31315), *UAS-Mad-RNAi* (BL-43183), *UAS-Medea-RNAi* (BL-43961), *UAS-rl-RNAi* (BL-34855), *UAS-sgg-RNAi* (BL-38293), *UAS-yki-RNAi* (BL-34067), and all deficiency (*Df*) lines (S1 Table). Of the two transgenic RNAi lines targeting *Mad*, the BL-31315 line (S4B and S4B’ Fig, Fig 6B) generated stronger effects than the BL-43183 line (e.g., Fig 5B) when expressed using the *ap-Gal4* driver. In addition, we tested the following mutant alleles of the Dpp signaling pathway: *Dad*^*j1E4*^*/TM3*, *Sb*^*1*^ (BL-10305), *Dad*^*MI04922*^*/TM3 Sb*^*1*^, *Ser*^*1*^ (BL-37913), *dpp*^*d6*^*/CyO* (BL-2062), *dpp*^*hr92*^*/SM6a* (BL-2069), *dpp*^*s11*^*/CyO* (BL-2065), *Mad*^*1-2*^*/CyO* (BL-7323), *Mad*^*12*^*/CyO* (BL-58785), *Mad*^*8-2*^*/CyO* (BL-7324), *Mad*^*k00237*^*/CyO* (BL-10474), *Mad*^*KG00581*^*/CyO* (BL-14578), *Medea*^*1*^*/TM3 Sb*^*1*^, *Ser*^*1*^ (BL-9033), *Medea*^*13*^*/TM3 Sb*^*1*^ (BL-7340), *tkv*^*7*^*/CyO* (BL-3242), and *tkv*^*k16713*^*/CyO* (BL-11191).

The following RNAi stocks, generated by the *Drosophila* TRiP project [91], were used to deplete the subunits of the Mediator complex: *UAS-Med1-RNAi* (BL-34662), *UAS-Med4-RNAi* (BL-34697), *UAS-Med6-RNAi/TM3 Sb*^*1*^ (BL-33743), *UAS-Med7-RNAi* (BL-34663), *UAS-Med8-RNAi* (BL-34926), *UAS-Med9-RNAi* (BL-33678), *UAS-Med10-RNAi* (BL-34031), *UAS-Med11-RNAi/TM3 Sb*^*1*^ (BL-34083), *UAS-Med12-RNAi* (BL-34588), *UAS-Med13-RNAi* (BL-34630), *UAS-Med14-RNAi* (BL-34575), *UAS-Med15-RNAi* (BL-32517), *UAS-Med16-RNAi* (BL-34012), *UAS-Med17-RNAi* (BL-34664), *UAS-Med18-RNAi* (BL-42634), *UAS-Med19-RNAi* (BL-33710), *UAS-Med20-RNAi* (BL-34577), *UAS-Med21-RNAi* (BL-34731), *UAS-Med22-RNAi* (BL-34573), *UAS-Med23-RNAi* (BL-34658), *UAS-Med24-RNAi* (BL-33755), *UAS-Med25-RNAi* (BL-42501), *UAS-Med26-RNAi* (BL-28572), *UAS-Med27-RNAi* (BL-34576), *UAS-Med28-RNAi/TM3 Sb*^*1*^ (BL-32459), *UAS-Med29-RNAi* (BL-57259), *UAS-Med30-RNAi/TM3 Sb*^*1*^ (BL-36711), and *UAS-Med31-RNAi* (BL-34574).

To facilitate the dominant modifier genetic screen and the subsequent analyses, we generated the following strains using the standard *Drosophila* genetics: “*w*^*1118*^*; nub-Gal4>UAS-Cdk8*^*+*^*/CyO*” (i.e., “*nub>Cdk8*^*+*^*/CyO*” line), “*w*^*1118*^*; nub-Gal4; UAS-Cdk8-RNAi*” (i.e., “*nub>Cdk8-i*” line), “*w*^*1118*^*; nub-Gal4; UAS-CycC-RNAi*” (i.e., “*nub>CycC-i*” line), “*w*^*1118*^*; nub-Gal4; UAS-Cdk8-RNAi CycC-RNAi*” (i.e., “*nub>Cdk8-i CycC-i*” line), and “*w*^*1118*^*; ap-Gal4*, *sal-lacZ/T(2*:*3)*”.

For the *Df* lines in the X chromosome, we crossed *Df* female virgins with males of with the “*nub>Cdk8*^*+*^*/CyO*”, “*nub>Cdk8-i*”, “*nub>CycC-i*”, or “*nub>Cdk8-i CycC-i”* stocks. For the *Df* lines in the second and third chromosomes, the *Df* males were crossed with female virgins of the afore-described stocks carrying the CDK8-specific phenotypes. The control crosses were performed using *w*^*1118*^ males and female virgins. For each of these crosses, the wing vein patterns in ~10 F1 females without any balancer chromosomes were inspected under dissecting microscopes for potential dominant modifications. With few exceptions (S1 Table), the wing vein phenotypes and dominant modifications are generally stereotypical with high penetrance. For instance, we crossed *Df(1)BSC531*, *w*^*1118*^*/FM7h* female virgins with “*w*^*1118*^*/Y; nub>Cdk8*^*+*^*/CyO*” males, and then scored F1 females with the following genotype: “*w*^*1118*^, *Df(1)BSC531/ w*^*1118*^*; nub>Cdk8*^*+*^*/+*”. Similarly, we crossed “*w*^*1118*^*; nub-Gal4; UAS-Cdk8-RNAi*” female virgins with “*Df(2R)Exel6064/CyO*” males, and then scored F1 females with the following genotype: “*w*^*1118*^*/+; nub-Gal4/Df(2R)Exel6064; UAS-Cdk8-RNAi/+*”. *Df* lines that caused lethality in F1 were considered as the enhancers.

### Adult *Drosophila* wing imaging

The wings from adult females were dissected onto slides, briefly washed using isopropanol, and then mounted in 50% Canada balsam diluted in isopropanol. Images were taken under 5X objective of a microscope (Leica DM2500) and then processed by Adobe Photoshop CS6 software.

### Immunocytochemistry

Wing discs from third instar larvae at the late wandering stage were dissected and fixed in 5% formaldehyde at room temperature for 30 minutes. After rinsing with PBS-Triton X-100 (0.2%), the samples were blocked in PBS-Triton X-100-NGS-BSA (PBS+0.2% Triton X-100+5% Normal Goat Serum+0.2% Bovine Serum Albumin) at room temperature for one hour. For immunostaining of *Drosophila* CDK8 and CycC, we used anti-dCDK8 (1:2000) and anti-dCycC (1:2000) antibodies [93–95], diluted in PBS-Triton X-100-NGS-BSA. Expression of the *lacZ* reporter expression was detected using an anti-β-galactosidase monoclonal antibody (1:50 in PBS-Triton X-100-NGS-BSA; obtained from the Developmental Studies Hybridoma Bank, DSHB-40-1a-s). C-terminal phosphorylated Mad (equivalent sites to human Smad3 S423+S425) was detected by anti-pSmad3 (1:500 in PBS-Triton X-100-NGS-BSA; purchased from Abcam, ab118825). Wing discs were incubated with these primary antibodies overnight at 4˚C on a rotator. After rinsing with PBS-Triton X-100, the discs were then incubated with the fluorophore conjugated secondary antibodies: goat anti-guinea pig (106-545-003), goat anti-mouse (115-545-003), or goat anti-rabbit (111-545-003) (all purchased from Jackson Immunological Laboratories). These secondary antibodies were diluted 1:1000 in PBS-Triton X-100-NGS-BSA, and incubated with the samples for one hour at room temperature. Discs were then stained with 1 μM DAPI at room temperature for 10 minutes, rinsed two more times with PBS-Triton X-100, and mounted in the Vectashield mounting media (Vector Laboratories, H-1000). Confocal images were taken with a Nikon Ti Eclipse confocal microscope system, with images processed using the Adobe Photoshop CS6 software.

Quantification of anti-β-galactosidase was performed with Nikon NIS software and Microsoft Excel: a single section of the wing discs was selected for the following quantification based on the DAPI channel, which indicates the cell nucleus are on the same focal plat. Three lines around 50μm long, 10–15μm apart, were drawn along the dorsal-ventral boundary. The line-scan profile of intensity for each line was calculated along each line (S5A and S5B Fig; genotype: *ap-Gal4*, *sal-lacZ/+; UAS-Cdk8-i/+*). The area below the intensity index profile represents the Sal-lacZ expression levels along the line (S5B Fig). To obtain the average intensity of dorsal or ventral compartment, the dorsal or ventral compartment index area was divided by the dorsal or ventral length of the line (S5C Fig). The intensity for three lines was normalized and averaged in dorsal and ventral compartments (S5C Fig, S5D Fig, S2 Table). Following this approach, five wing discs for each genotype were analyzed to quantify the expression of Sal-lacZ in dorsal and ventral compartments, and statistical significance was calculated using Student’s one-tailed *t*-test (S5E Fig, S2 Table).

To validate the afore described quantification method, we also measured the signaling intensity by selecting 20x20μm squares in the dorsal and the ventral compartments of the same wing disc using the Nikon NIS software (S6A and S6B Fig; genotype: *ap-Gal4*, *sal-lacZ/+; UAS-Med15-i/+*). We then calculated the dorsal to ventral ratios of the signal intensities of three different discs (S6B Fig), followed by statistical analyses using the Student’s one-tailed *t*-test (S6C and S6D Fig). We obtained similar results to the quantification based on the line profiles as described above.

### GST-pull down assay

Full-length CDK8 fused with a N-terminal GST tag was described previously [36]. The primers *Mad-5*.*1* (F: 5’-caccATGGACACCGACGATGTGGA-3’) and *Mad-3*.*3* (F: 5’-ctaTTAGGATACCGAACTAATTG-3’) were used for full-length Mad (AA1-455), *Mad-5*.*1* and *Mad-3*.*1* (F: 5’-ctaCGGGAGCACCGGACTCTCCA-3’) were used for a “Mad-N1” fragment (AA1-150) that contains MH1 domain (AA10-133), *Mad-5*.*1* and *Mad-3*.*2* (F: 5’-ctaATCCTCCGAGGGACTGTAGG-3’) were used for the “Mad-N2” fragment (AA1-230) that contains the MH1 domain and part of the linker region, *Mad-5*.*2* (F: 5’-caccatgCCAGTACTCGTTCCTCGCCA-3’) and *Mad-3*.*3* were used for the “Mad-C2” fragment (AA151-455) that contains the MH2 domain (AA255-455) and part of the linker region, and *Mad-5*.*3* (F: 5’-caccatgGGCAACTCCAACAATCCGAA-3’) and *Mad-3*.*3* were for the “Mad-C1” fragment (AA231-455) that contains the MH2 domain. These coding sequences were amplified from a cDNA clone of the *Mad* gene (LD12679) using PrimeStar Max premix (Takara, R045A). The amplified products were inserted into the pENTR/D-TOPO vector (ThermoFisher, K240020) and recombined into the pDEST17 vector (N-terminal 6XHis tag) using the Gateway LR Clonase II Enzyme mix (ThermoFisher, 11791100) in *E*. *coli* strain DH5α. The constructs were transformed to *E*. *coli* strain Rosetta, received from Craig Kaplan, for protein expression using standard protocols.

GST or GST-CDK8 was purified with Glutathione Sepharose 4B (GE Healthcare, 17-0756-01) beads with standard purification protocol. After a final wash, the buffer was replaced by the GST pull-down buffer (20mM Tris-HCl pH 7.5, 10mM MgCl_2_, 100mM NaCl, 1mM DTT, 0.1% NP-40). His-tagged Mad fragments were extracted from the pull-down buffer by sonication. 50μL GST or GST-CDK8 coated beads (0.5–1μg protein) was mixed with 500μL of Mad fragments cell lysate and incubated at 4°C for 3 hours. These samples were then washed with 1mL pull-down buffer at 4°C for 5 times, 1 minute each. The interaction was detected by Western Blot with the primary antibody, anti-His (1:3000; Sigma, H1029), and the secondary antibody, anti-mouse (1:2000; Jackson Immunological Laboratories, 115-035-174).

### Yeast two-hybrid (Y2H) assay

Full-length CDK8 was amplified from a pBS-CDK8 cDNA clone using primers *CDK8-5*.*1* (F: 5’-caccATGGACTACG ATTTCAAGAT-3’) and *CDK8-3*.*1* (F: 5’-TCAGTTGAAGCGCTGGAAGT-3’), and then inserted into the pENTR/D-TOPO vector. The Gateway LR Clonase II Enzyme mix was used to recombine CDK8 cDNA into the pGADT7-GW (prey) vector, a gift from Yuhai Cui (Addgene plasmid # 61702) [96]. The linker region of Mad was amplified with *Mad-5*.*2* and *Mad-3*.*2* primers from a cDNA clone of the *Mad* gene (LD12679) using PrimeStar Max premix and inserted into the pENTR/D-TOPO vector. All pENTR Mad fragments were recombined into the pGBKT7-GW (bait) vector, a gift from Yuhai Cui (Addgene plasmid # 61703) [96], using the Gateway LR Clonase II Enzyme mix. The Y2H assay was performed using the AH109 yeast strain, as described previously [96].

### Statistical analysis

Standard deviation and Student’s one-tailed *t*-tests were performed using Microsoft Excel. Statistical significance (* *p*<0.05; ** *p*<0.01; *** *p*<0.001) was shown in figures and all error bars indicate standard deviation.

## Supporting information

S1 Fig Effects of CDK8 on the size of wings, cell number, and cell sizes. (A) A normal wing; (B) high magnification of an L3-L4 intervein region showing the hairs in wing cells. (C) Quantification of wing sizes, total cell numbers, and hair density (reflecting cell sizes) in the control (black bars, genotype: “*nub-Gal4/+; +*”), CDK8- and CycC-depleted (blue, “*nub-Gal4/+; UAS-Cdk8-RNAi CycC-RNAi/+*”), and CDK8-overexpressing (red, “*nub-Gal4 UAS-Cdk8*^*+*^*/+; +*”) wings. One-sided *t*-tests were used to determine the statistical significance of the differences.
(TIF)

S2 Fig Effects of CDK8 on the wing morphology with *ap-Gal4*. Representative adult wings of (A) *w*^*1118*^; (B) *CyO/+;* (C) *ap-Gal4/+; UAS-Cdk8-RNAi CycC-RNAi/+;* (D) *ap-Gal4/UAS-Cdk8*^*+*^
(TIF)

S3 Fig Effects of Dpp signaling pathway components on *nub-Gal4*. Representative confocal images of RFP signal of the wing pouch area of discs of the following genotypes: (A) *nub-Gal>RFP/+*; (B) *nub-Gal>RFP/Mad*^*1-2*^; and (C) *nub-Gal>RFP/+; Dad*^*J14E*^*/+*. At least five discs were examined for each genotype. All these images were taken at the same settings for fixations, staining, and confocal imaging.
(TIF)

S4 Fig Validation of the *sal-lacZ* reporter. Representative confocal images of anti-β-Gal stainings of the wing pouch area of discs of the following genotypes: (A) *ap-Gal4*, *sal-lacZ/+; UAS-dpp-RNAi/+*; (B) *ap-Gal4*, *sal-lacZ/+; UAS-Mad-RNAi*/+ (BL-31315); and (B’) merge image of DAPI (blue) and anti-β-gal (green) channel of *ap-Gal4*, *sal-lacZ/+; UAS-Mad-RNAi/ +* (BL-31315). At least five discs were examined for each genotype.
(TIF)

S5 Fig Quantification of the *sal-lacZ* expression. (A) Three lines were drawn across the dorsal-ventral compartment boundary within the wing pouch area to calculate the intensity index profile; genotype: *ap-Gal4*, *sal-lacZ/+; UAS-Cdk8-i/+*. (B) An example of the index profile of one line, measured area below the index profile, and its length. (C) Average and normalization of average intensity of the anti-β-Gal staining in the dorsal and ventral compartments of a wing disc. (D) Average of three lines. (E) Student’s *t*-test was used to compare the Sal-lacZ expression levels in the dorsal and ventral compartments of five discs of the same genotype. See Materials and Methods for more details.
(TIF)

S6 Fig An alternative method to quantify the *sal-lacZ* expression. (A) 20x20μm squares were drawn in both dorsal and ventral compartments; genotype: *ap-Gal4*, *sal-lacZ/+; UAS-Med15-i/+*). (B) Mean intensity of the anti-β-Gal staining of three different discs within the taken squares was given. Dorsal to ventral ratio of each disc was calculated. Student’s *t*-test was used to compare the Sal-lacZ expression levels ratio between different genotypes (C), and plotted as column chart (D). See Materials and Methods for more details.
(TIF)

S7 Fig Effects of depleting subunits of the CKM and CDK9 on *ap-Gal4*, and effect of depleting Ap on *sal-lacZ* expression. Representative confocal images of GFP (green) and DAPI (blue) signal of the wing pouch area of discs of the following genotypes: (A) *ap-Gal>GFP/+*; (B) *ap-Gal4>GFP/+; UAS-Cdk8-i*,*CycC-i/+*; (D) *ap-Gal4>GFP/+; UAS-Cdk9-i/+*; (E) *ap-Gal4>GFP/+;UAS-Med12-i/+*; (F) *ap-Gal4>GFP/+;UAS-Med13-i/+*; (C) Confocal images of anti-β-Gal staining of wing discs of *ap-Gal4*,*sal-lacZ/+; UAS-ap-i/+*. At least five discs were examined for each genotype.
(TIF)

S8 Fig Depletion of CDK8 or CycC does not affect the levels of p-Mad. Representative confocal images of anti-p-Mad staining of wing discs from the following genotypes: (A) *ap-Gal4*, *sal-lacZ/+* (control); (B) *ap-Gal4*, *sal-lacZ/+; UAS-Cdk8-i/+*; (C) *ap-Gal4*, *sal-lacZ/+; UAS-CycC-i*; and (D) *ap-Gal4*, *sal-lacZ/+; UAS-Cdk8-i CycC-i*. At least five discs were examined for each genotype. Scale bar in D: 25μm.
(TIF)

S9 Fig Additional results from the Y2H assay. Full-length (FL) Mad, Mad-C2 fragment or CDK8 proteins as the bait are able to auto-activate in this assay. Refer the figure legend in Fig 5 and the Materials and Methods for more details.
(TIF)

S10 Fig Depletion of dedicated Mediator subunits strongly disrupted wing disc morphology. Representative confocal images of anti-β-Gal staining of wing discs of the following genotypes: (A) *ap-Gal4*, *sal-lacZ/+; UAS-Med8-RNAi/+*; (B) *ap-Gal4*, *sal-lacZ/+; UAS-Med14-RNAi/+*; (C) *ap-Gal4*, *sal-lacZ/+; UAS-Med16-RNAi/+*; (D) *ap-Gal4*, *sal-lacZ/+; UAS-Med17-RNAi/+*; (E) *ap-Gal4*, *sal-lacZ/+; UAS-Med21-RNAi/+*; (F) in *ap-Gal4*, *sal-lacZ/+; UAS-Med22-RNAi/+*; and (G) *ap-Gal4/+; vgQE-lacZ/UAS-Med21-RNAi*. At least five discs were examined for each genotype. Scale bar in F: 25μm.
(TIF)

S1 Table Results of 490 deficiency (*Df*) lines tested for potential dominant modification of vein phenotypes caused by altered levels of CDK8 or CycC. (XLSX)

S2 Table Quantification of the Sal-lacZ expression. Normalization of average intensity of the anti-β-Gal staining in the dorsal and ventral compartments of wing discs. Five wing discs were analyzed for each genotype, except *UAS-Cdk7-i* and *UAS-sgg-i* (N = 3). Student’s *t*-test was used to compare the Sal-lacZ expression levels in the dorsal and ventral compartments of five discs of the same genotype. See Materials and Methods for more details.
(XLSX)
