# Supplementary figures and images for: The Mediator CDK8-Cyclin C complex modulates Dpp signaling in Drosophila by stimulating Mad-dependent transcription
Source: PLoS Genet. 2020 May 28;16(5):e1008832. doi: 10.1371/journal.pgen.1008832 (PMC7282676; doi:10.1371/journal.pgen.1008832)

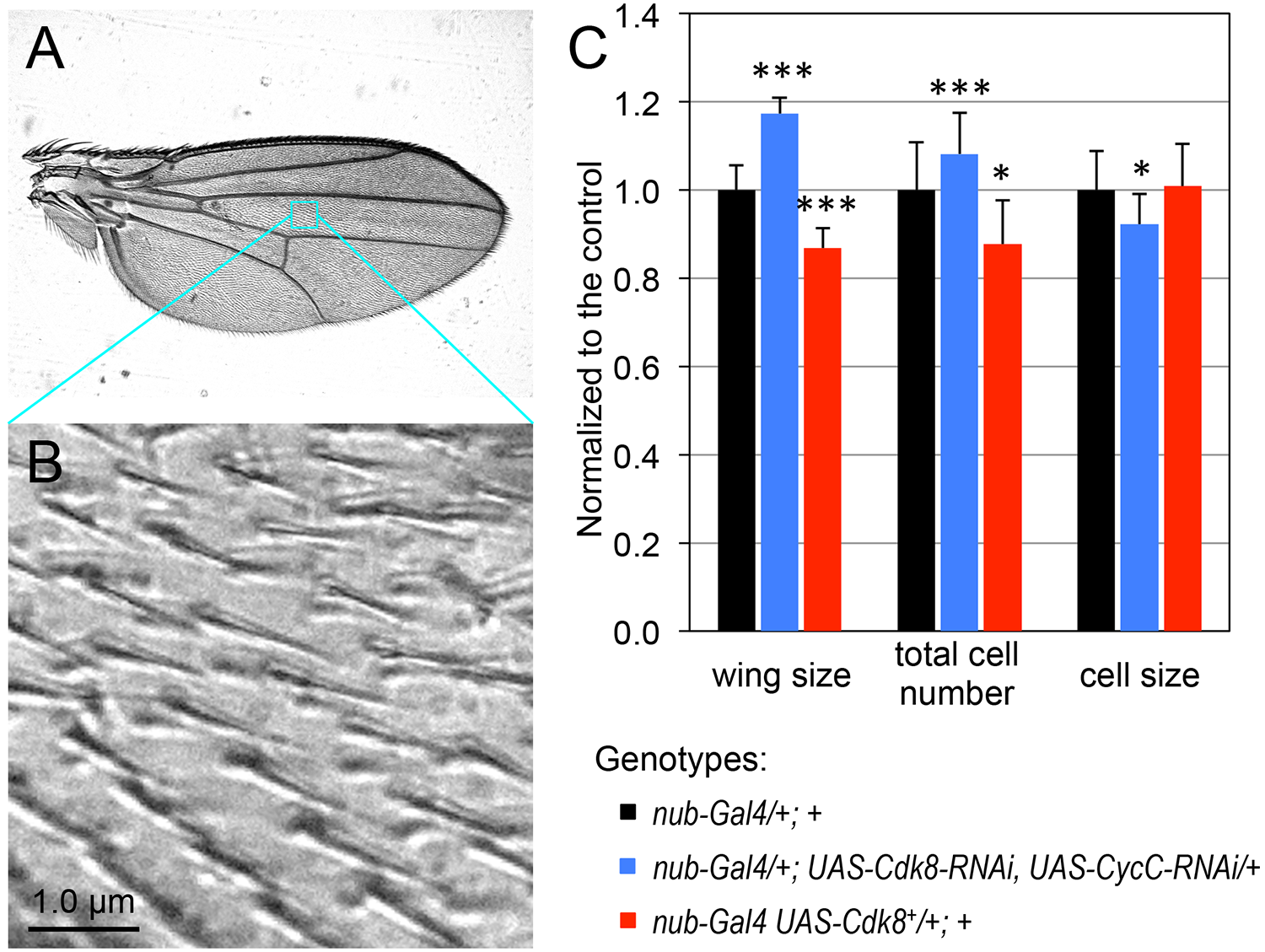

Supplement: S1 Fig — (A) A normal wing; (B) high magnification of an L3-L4 intervein region showing the hairs in wing cells. (C) Quantification of wing sizes, total cell numbers, and hair density (reflecting cell sizes) in the control (black bars, genotype: “nub-Gal4/+; +”), CDK8- and CycC-depleted (blue, “nub-Gal4/+; UAS-Cdk8-RNAi CycC-RNAi/+”), and CDK8-overexpressing (red, “nub-Gal4 UAS-Cdk8+/+; +”) wings. One-sided t-tests were used to determine the statistical significance of the differences. (TIF) [file pgen.1008832.s001.tif]

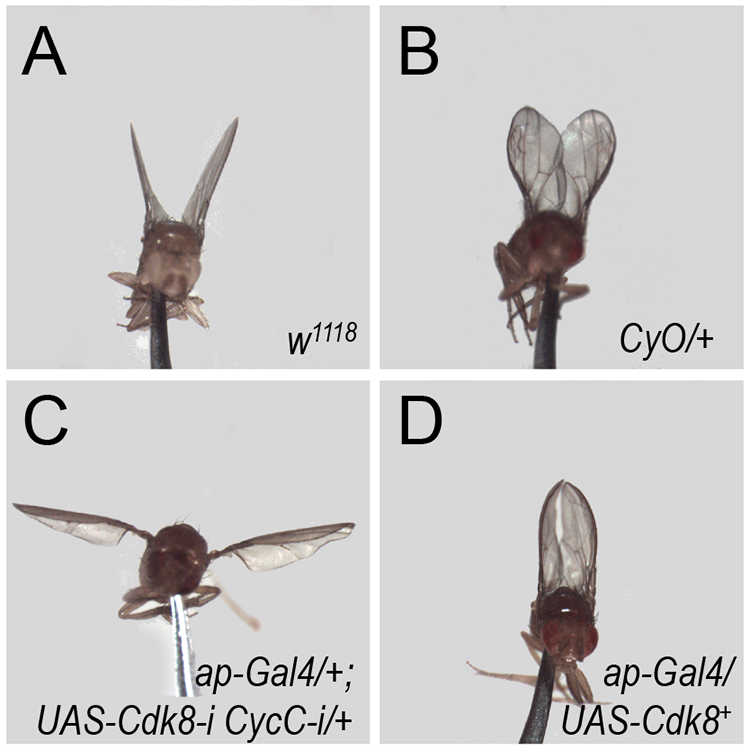

Supplement: S2 Fig — Representative adult wings of (A) w1118; (B) CyO/+; (C) ap-Gal4/+; UAS-Cdk8-RNAi CycC-RNAi/+; (D) ap-Gal4/UAS-Cdk8+ (TIF) [file pgen.1008832.s002.tif]

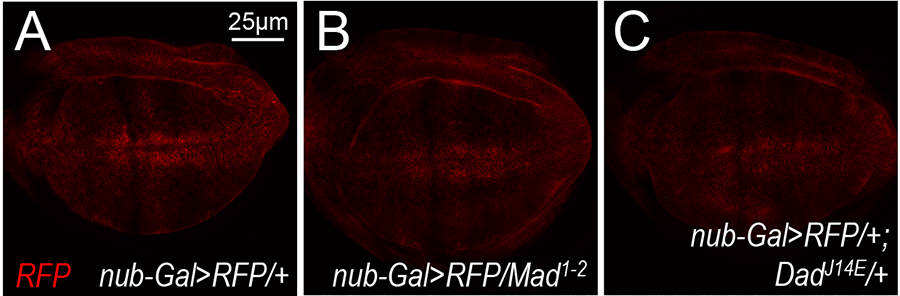

Supplement: S3 Fig — Representative confocal images of RFP signal of the wing pouch area of discs of the following genotypes: (A) nub-Gal>RFP/+; (B) nub-Gal>RFP/Mad1-2; and (C) nub-Gal>RFP/+; DadJ14E/+. At least five discs were examined for each genotype. All these images were taken at the same settings for fixations, staining, and confocal imaging. (TIF) [file pgen.1008832.s003.tif]

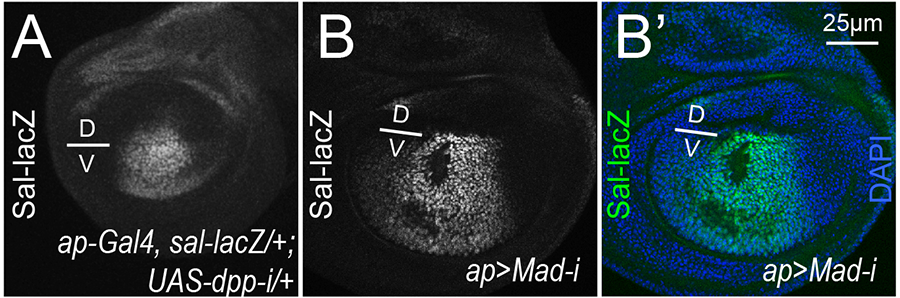

Supplement: S4 Fig — Representative confocal images of anti-β-Gal stainings of the wing pouch area of discs of the following genotypes: (A) ap-Gal4, sal-lacZ/+; UAS-dpp-RNAi/+; (B) ap-Gal4, sal-lacZ/+; UAS-Mad-RNAi/+ (BL-31315); and (B’) merge image of DAPI (blue) and anti-β-gal (green) channel of ap-Gal4, sal-lacZ/+; UAS-Mad-RNAi/ + (BL-31315). At least five discs were examined for each genotype. (TIF) [file pgen.1008832.s004.tif]

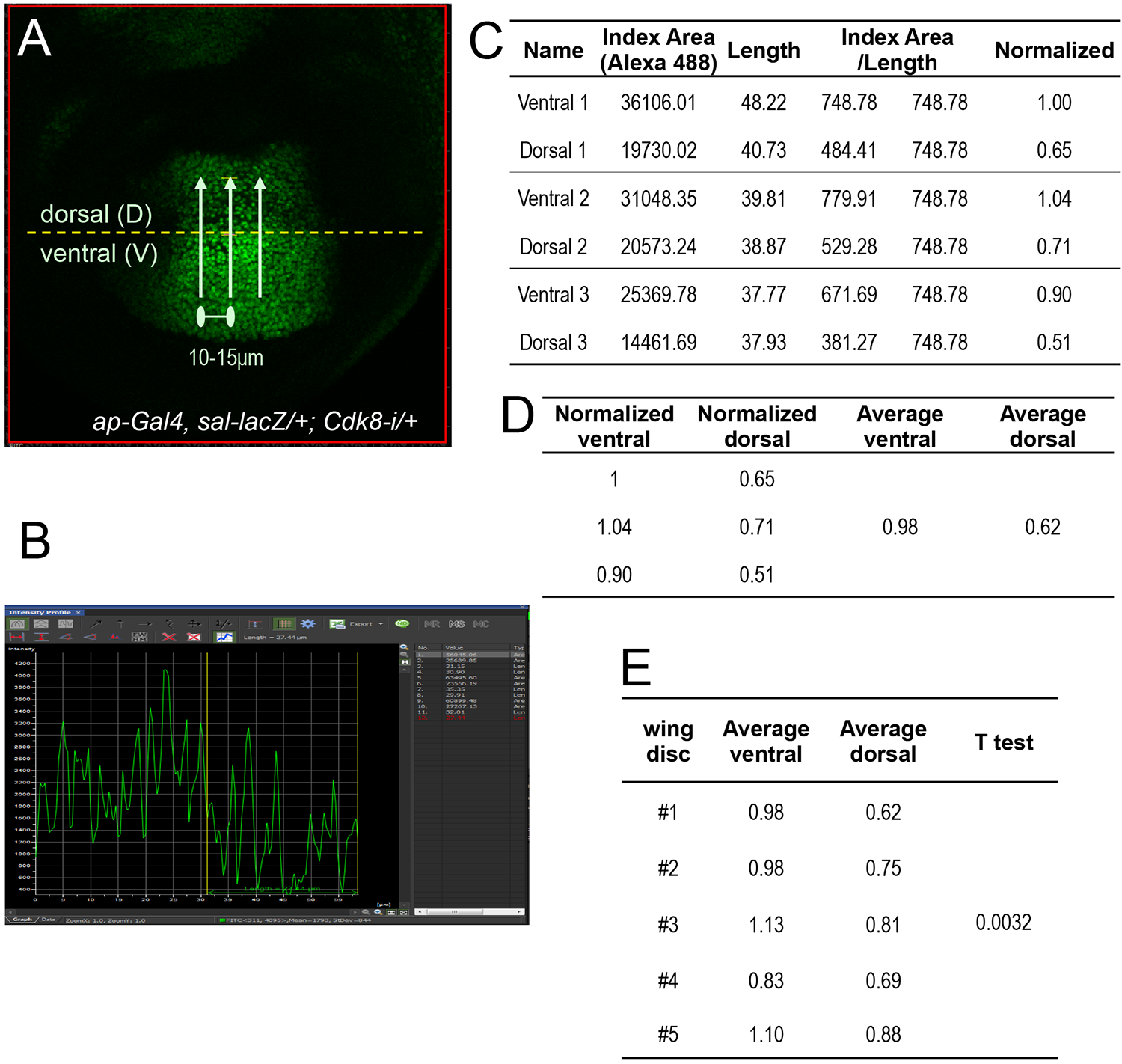

Supplement: S5 Fig — (A) Three lines were drawn across the dorsal-ventral compartment boundary within the wing pouch area to calculate the intensity index profile; genotype: ap-Gal4, sal-lacZ/+; UAS-Cdk8-i/+. (B) An example of the index profile of one line, measured area below the index profile, and its length. (C) Average and normalization of average intensity of the anti-β-Gal staining in the dorsal and ventral compartments of a wing disc. (D) Average of three lines. (E) Student’s t-test was used to compare the Sal-lacZ expression levels in the dorsal and ventral compartments of five discs of the same genotype. See Materials and Methods for more details. (TIF) [file pgen.1008832.s005.tif]

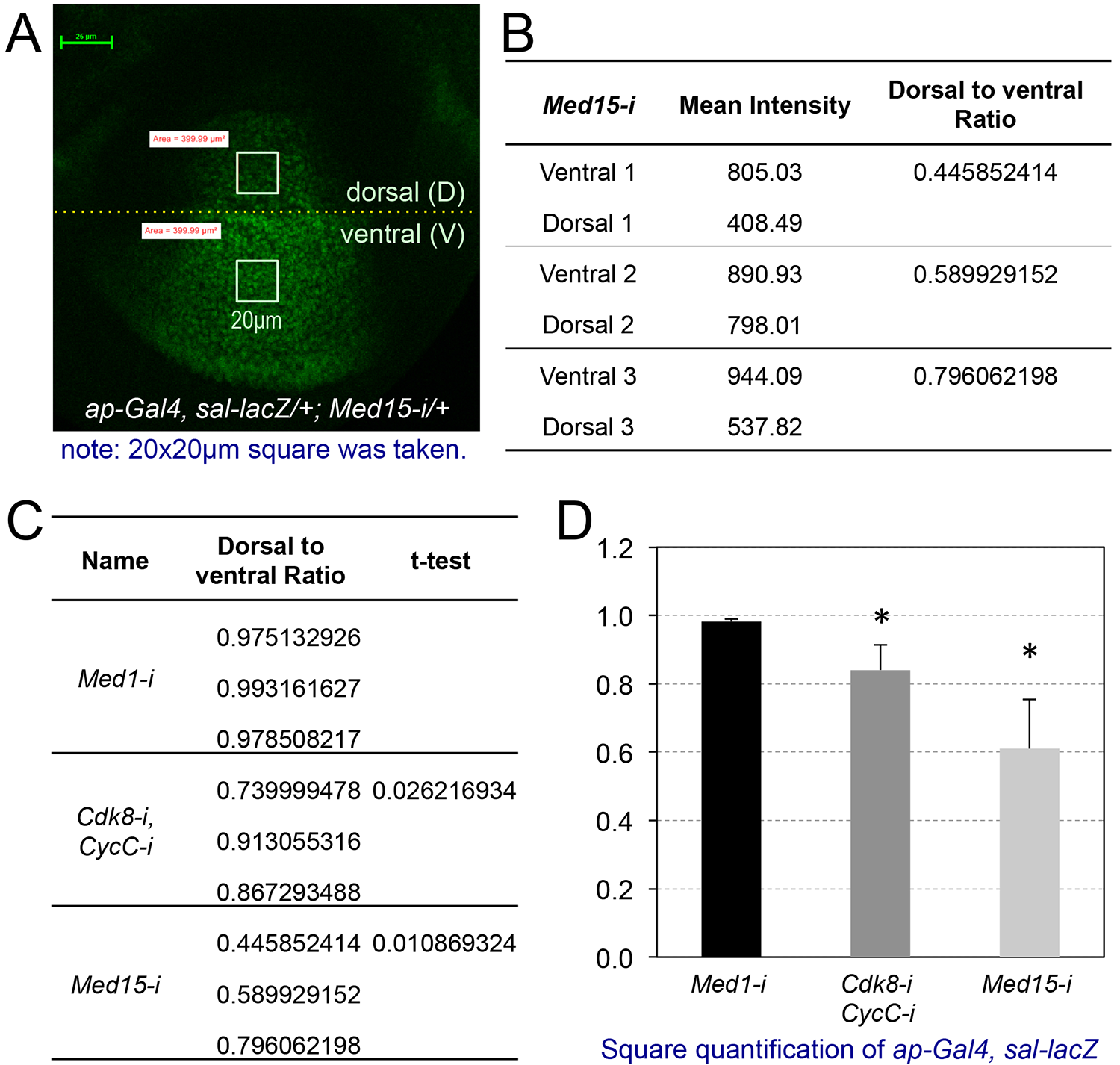

Supplement: S6 Fig — (A) 20x20μm squares were drawn in both dorsal and ventral compartments; genotype: ap-Gal4, sal-lacZ/+; UAS-Med15-i/+). (B) Mean intensity of the anti-β-Gal staining of three different discs within the taken squares was given. Dorsal to ventral ratio of each disc was calculated. Student’s t-test was used to compare the Sal-lacZ expression levels ratio between different genotypes (C), and plotted as column chart (D). See Materials and Methods for more details. (TIF) [file pgen.1008832.s006.tif]

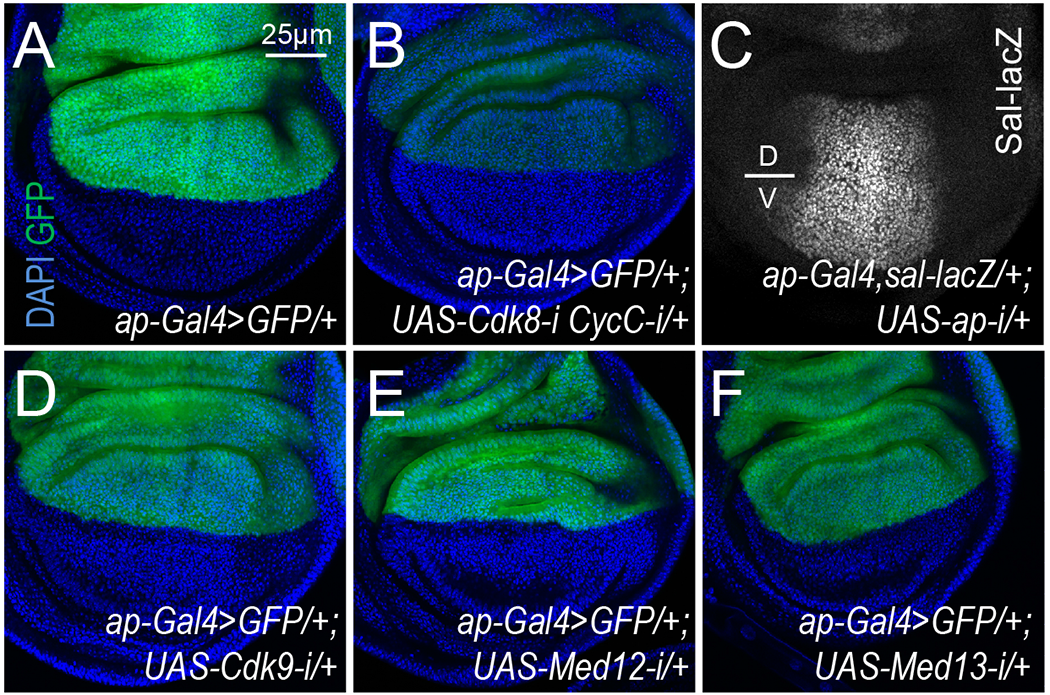

Supplement: S7 Fig — Representative confocal images of GFP (green) and DAPI (blue) signal of the wing pouch area of discs of the following genotypes: (A) ap-Gal>GFP/+; (B) ap-Gal4>GFP/+; UAS-Cdk8-i,CycC-i/+; (D) ap-Gal4>GFP/+; UAS-Cdk9-i/+; (E) ap-Gal4>GFP/+;UAS-Med12-i/+; (F) ap-Gal4>GFP/+;UAS-Med13-i/+; (C) Confocal images of anti-β-Gal staining of wing discs of ap-Gal4,sal-lacZ/+; UAS-ap-i/+. At least five discs were examined for each genotype. (TIF) [file pgen.1008832.s007.tif]

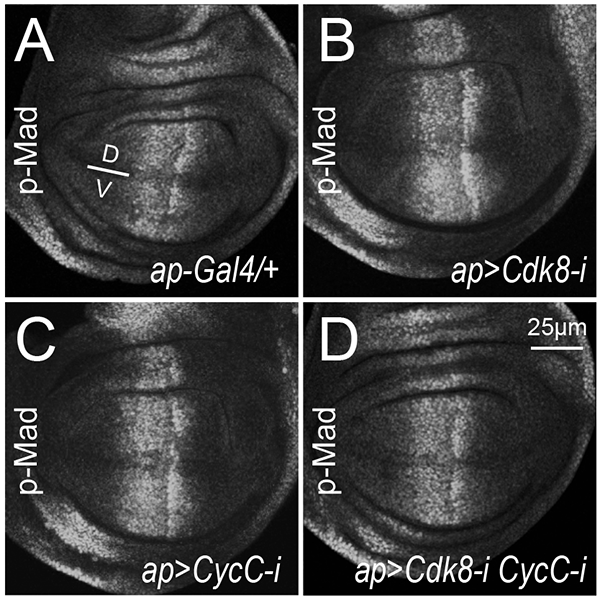

Supplement: S8 Fig — Representative confocal images of anti-p-Mad staining of wing discs from the following genotypes: (A) ap-Gal4, sal-lacZ/+ (control); (B) ap-Gal4, sal-lacZ/+; UAS-Cdk8-i/+; (C) ap-Gal4, sal-lacZ/+; UAS-CycC-i; and (D) ap-Gal4, sal-lacZ/+; UAS-Cdk8-i CycC-i. At least five discs were examined for each genotype. Scale bar in D: 25μm. (TIF) [file pgen.1008832.s008.tif]

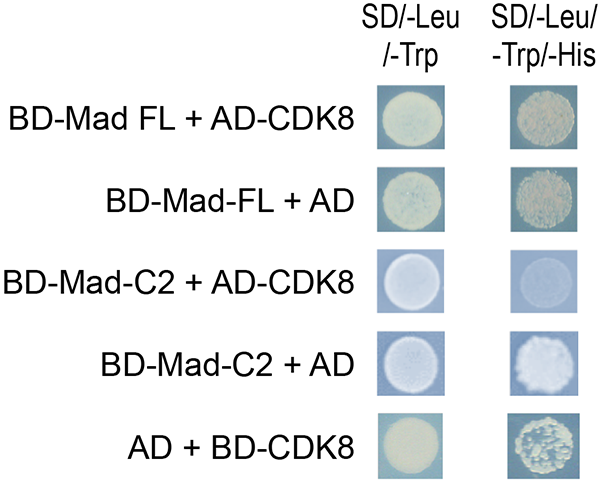

Supplement: S9 Fig — Full-length (FL) Mad, Mad-C2 fragment or CDK8 proteins as the bait are able to auto-activate in this assay. Refer the figure legend in Fig 5 and the Materials and Methods for more details. (TIF) [file pgen.1008832.s009.tif]

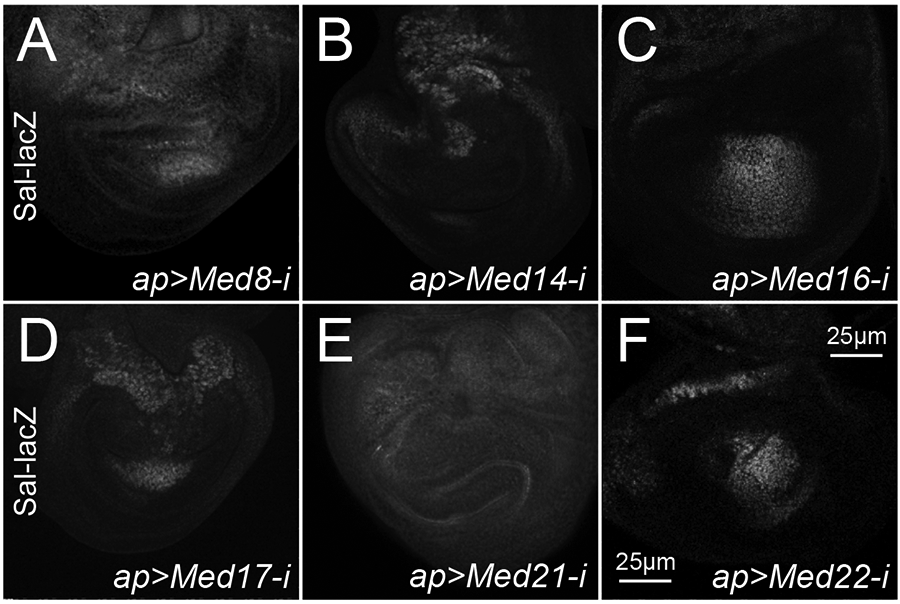

Supplement: S10 Fig — Representative confocal images of anti-β-Gal staining of wing discs of the following genotypes: (A) ap-Gal4, sal-lacZ/+; UAS-Med8-RNAi/+; (B) ap-Gal4, sal-lacZ/+; UAS-Med14-RNAi/+; (C) ap-Gal4, sal-lacZ/+; UAS-Med16-RNAi/+; (D) ap-Gal4, sal-lacZ/+; UAS-Med17-RNAi/+; (E) ap-Gal4, sal-lacZ/+; UAS-Med21-RNAi/+; (F) in ap-Gal4, sal-lacZ/+; UAS-Med22-RNAi/+; and (G) ap-Gal4/+; vgQE-lacZ/UAS-Med21-RNAi. At least five discs were examined for each genotype. Scale bar in F: 25μm. (TIF) [file pgen.1008832.s010.tif]
